# Multiple lines of evidence for disruption of nuclear lamina and nucleoporins in FUS amyotrophic lateral sclerosis

**DOI:** 10.1093/brain/awae224

**Published:** 2024-09-23

**Authors:** Kensuke Okada, Daisuke Ito, Satoru Morimoto, Chris Kato, Yuki Oguma, Hitoshi Warita, Naoki Suzuki, Masashi Aoki, Junko Kuramoto, Reona Kobayashi, Munehisa Shinozaki, Masahito Ikawa, Jin Nakahara, Shinichi Takahashi, Yoshinori Nishimoto, Shinsuke Shibata, Hideyuki Okano

**Affiliations:** Department of Neurology, Keio University School of Medicine, Tokyo 160-8582, Japan; Department of Physiology, Keio University School of Medicine, Tokyo 160-8582, Japan; Keio University iPS Cell Research Center for Intractable Neurological Diseases (KiND), Keio University Global Research Institute, Tokyo 108-0073, Japan; Department of Neurology, Keio University School of Medicine, Tokyo 160-8582, Japan; Department of Physiology, Keio University School of Medicine, Tokyo 160-8582, Japan; Keio University iPS Cell Research Center for Intractable Neurological Diseases (KiND), Keio University Global Research Institute, Tokyo 108-0073, Japan; Memory Center, Keio University School of Medicine, Tokyo 160-8582, Japan; Department of Physiology, Keio University School of Medicine, Tokyo 160-8582, Japan; Keio University iPS Cell Research Center for Intractable Neurological Diseases (KiND), Keio University Global Research Institute, Tokyo 108-0073, Japan; Keio University Regenerative Medicine Research Center, Kanagawa, 210-0821, Japan; Division of Neurodegenerative Disease Research, Tokyo Metropolitan Institute for Geriatrics and Gerontology, Tokyo, 173-0015, Japan; Department of Physiology, Keio University School of Medicine, Tokyo 160-8582, Japan; Keio University Regenerative Medicine Research Center, Kanagawa, 210-0821, Japan; Division of Neurodegenerative Disease Research, Tokyo Metropolitan Institute for Geriatrics and Gerontology, Tokyo, 173-0015, Japan; Department of Physiology, Keio University School of Medicine, Tokyo 160-8582, Japan; Keio University Regenerative Medicine Research Center, Kanagawa, 210-0821, Japan; Department of Neurology, Tohoku University Graduate School of Medicine, Sendai, 980-8574, Japan; Department of Neurology, Tohoku University Graduate School of Medicine, Sendai, 980-8574, Japan; Department of Neurology, Tohoku University Graduate School of Medicine, Sendai, 980-8574, Japan; Department of Pathology, Keio University School of Medicine, Tokyo, 160-8582, Japan; Department of Physiology, Keio University School of Medicine, Tokyo 160-8582, Japan; Department of Physiology, Keio University School of Medicine, Tokyo 160-8582, Japan; Keio University Regenerative Medicine Research Center, Kanagawa, 210-0821, Japan; Research Institute for Microbial Diseases, Osaka University, Suita, Osaka, 565-0871, Japan; Department of Neurology, Keio University School of Medicine, Tokyo 160-8582, Japan; Keio University iPS Cell Research Center for Intractable Neurological Diseases (KiND), Keio University Global Research Institute, Tokyo 108-0073, Japan; Department of Physiology, Keio University School of Medicine, Tokyo 160-8582, Japan; Keio University iPS Cell Research Center for Intractable Neurological Diseases (KiND), Keio University Global Research Institute, Tokyo 108-0073, Japan; Keio University Regenerative Medicine Research Center, Kanagawa, 210-0821, Japan; Department of Neurology and Stroke, Saitama Medical University International Medical Center, Saitama, 350-1298, Japan; Department of Neurology, Keio University School of Medicine, Tokyo 160-8582, Japan; Department of Physiology, Keio University School of Medicine, Tokyo 160-8582, Japan; Division of Microscopic Anatomy, Graduate School of Medical and Dental Sciences, Niigata University, Niigata, 951-8510, Japan; Department of Physiology, Keio University School of Medicine, Tokyo 160-8582, Japan; Keio University iPS Cell Research Center for Intractable Neurological Diseases (KiND), Keio University Global Research Institute, Tokyo 108-0073, Japan; Keio University Regenerative Medicine Research Center, Kanagawa, 210-0821, Japan; Division of Neurodegenerative Disease Research, Tokyo Metropolitan Institute for Geriatrics and Gerontology, Tokyo, 173-0015, Japan; Laboratory for Marmoset Models of Neural Diseases, RIKEN Center for Brain Science, Saitama, 351-0198, Japan

**Keywords:** FUS, amyotrophic lateral sclerosis, nuclear lamina, nuclear pore complex

## Abstract

Advanced pathological and genetic approaches have revealed that mutations in fused in sarcoma/translated in liposarcoma (FUS/TLS), which is pivotal for DNA repair, alternative splicing, translation and RNA transport, cause familial amyotrophic lateral sclerosis (ALS). The generation of suitable animal models for ALS is essential for understanding its pathogenesis and developing therapies. Therefore, we used CRISPR-Cas9 to generate *FUS*-ALS mutation in the non-classical nuclear localization signal (NLS), H517D (mouse position: H509D) and genome-edited mice.

*Fus* ^WT/H509D^ mice showed progressive motor impairment (accelerating rotarod and DigiGait system) with age, which was associated with the loss of motor neurons and disruption of the nuclear lamina and nucleoporins and DNA damage in spinal cord motor neurons. We confirmed the validity of our model by showing that nuclear lamina and nucleoporin disruption were observed in lower motor neurons differentiated from patient-derived human induced pluripotent stem cells (hiPSC-LMNs) with *FUS*-H517D and in the post-mortem spinal cord of patients with ALS. RNA sequence analysis revealed that most nuclear lamina and nucleoporin-linking genes were significantly decreased in *FUS*-H517D hiPSC-LMNs.

This evidence suggests that disruption of the nuclear lamina and nucleoporins is crucial for ALS pathomechanisms. Combined with patient-derived hiPSC-LMNs and autopsy samples, this mouse model might provide a more reliable understanding of ALS pathogenesis and might aid in the development of therapeutic strategies.

## Teaser

Using genome-edited mice, patient-derived iPSC-motor neurons and post-mortem spinal cord, Okada *et al.* reveal disruption of the nuclear membrane and nucleoporins in *FUS*-ALS. Aberrant distribution of FUS protein disrupts the nuclear membrane, nucleoporins, and RNA quality control, leading to impaired nuclear-cytoplasmic trafficking.

## Introduction

Amyotrophic lateral sclerosis (ALS) is a fatal neurological disease characterized by degeneration of motor neurons (MNs), resulting in symptoms of muscle weakness and atrophy and death from respiratory failure within 3–5 years of symptom onset.^1^ Riluzole, edaravone have been approved by the USA Food and Drug Administration; however, their efficacy is limited, and no effective therapy has been identified to halt the progression of ALS. Approximately 10% of patients with ALS have a family history of ALS, and several gene mutations (including *C9orf72*, *SOD1*, *TARDBP* and *FUS*) have been reported.^2^ Fused in sarcoma (*FUS*) has been reported in ∼3% of familial ALS and 1% of sporadic ALS and is the second most common ALS mutated gene in Japan, following *SOD1.*^3,4^ However, >30% of juvenile ALS patients are reported to have *FUS* gene mutations.^5^ FUS is an RNA-binding protein that is normally localized in the nucleus, but has an altered cytoplasmic localization in ALS.^6,7^ ALS mutations in *FUS* cluster in the non-classical nuclear localization signal (NLS) at the C-terminus, which directly prevent nuclear localization, resulting in aberrant cytoplasmic accumulation of FUS protein in a pathological cascade leading to the ALS phenotype.^8,9^

There are numerous reports on the generation of an ALS mouse model targeting the *Fus* gene. *Fus* knockout mice do not show MN deficits, whereas transgenic mice with *Fus* mutant genes show ALS- and frontotemporal dementia (FTD)-like phenotypes.^10-13^ However, overexpression of the ALS-related gene beyond the physiological range might cause unwanted artefacts. In addition, there are questions about the complications of variation in copy number and insertion site in transgenic mice. Studies have reported that 5%–10% of transgene integration events result in an undesired phenotype owing to the disruption of endogenous gene loci by transgenes.^14-16^

To address this issue, we generated *FUS*-ALS genome-edited mice using the CRISPR-Cas9 system. We selected *FUS*^H517D^, which leads to the classic *FUS*-ALS phenotype with neck or proximal upper limb weakness as the first symptom.^17^ This mutation replaces the basic amino acid histidine in the NLS with the acidic amino acid aspartic acid, resulting in a large charge change and conformational change that is expected to produce prominent phenotypes. In our previous study, induced pluripotent stem cell (iPSC)-derived MNs from patients with ALS and the *FUS*^H517D^ mutation exhibited several neurodegenerative phenotypes, including FUS mislocalization into the cytoplasm and aberrant stress granule formation in stress conditions and cellular vulnerability.^18^

Here, we report the generation and characterization of *Fus*^WT/H509D^ genome-edited mice as a new animal model of ALS. *Fus*^WT/H509D^ genome-edited mice exhibited motor dysfunction with ageing, FUS mislocalization to the cytoplasm without inclusions, and disruption of the nuclear lamina and nucleoporins in the MNs of the spinal cord. In addition, we confirmed disruption of the nuclear lamina and nucleoporins in human induced pluripotent stem cell (hiPSC)-derived MNs with the *FUS*^H517D^ mutation, corresponding to *Fus*^H509D^ in mice, and post-mortem spinal cord from patients with ALS. Combined with hiPSC-MNs and human samples, this mouse model is expected to provide satisfactory results for the investigation of ALS pathomechanisms and the development of therapeutics.

## Materials and methods

### Generation of *Fus*^H509D^ mutant mice

**Figure 1:**
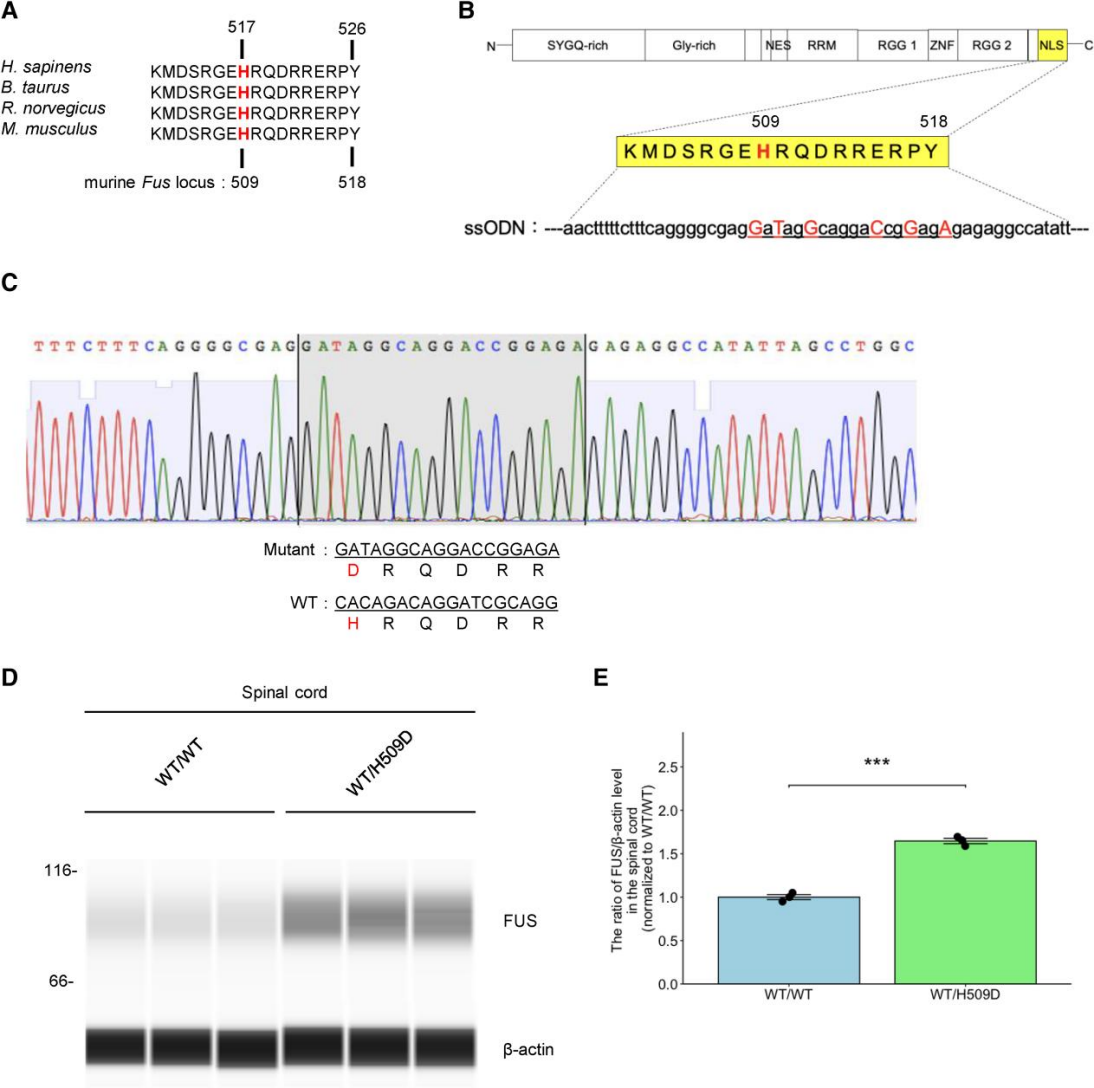
**Generation of *Fus*^WT/H509D^ genome-edited mice.** (**A**) Alignment of amino acids in the nuclear localization signal (NLS) of fused in sarcoma (FUS) in different mammalian species. The NLS is highly conserved between humans and rodents. Red indicates the H517 residue. (**B**) Design of single-stranded oligodeoxynucleotides (ssODN) in the CRISPR-Cas9-based genome editing approach. Silent mutations without amino acid mutations were inserted (in bold and red) to prevent re-cleavage by the guide ribonucleic acid (gRNA) after genome editing. The mouse position corresponding to human H517 is H509. (**C**) Sequence of NLS of *Fus* in wild-type (WT) and *Fus*^WT/H509D^ mice. (**D**) Expression of FUS in the mouse spinal cord. Tissues from wild-type and *Fus*^WT/H509D^ mice at 25 months were blotted using an FUS antibody. β-Actin was used as the loading control. (**E**) Ratio of FUS/β-actin in the spinal cord from wild-type and *Fus*^WT/H509D^ mice at 25 months (overlaid bar graph and dot plot). Data are presented as the mean ± SEM. Student’s *t*-test was used to calculate statistical significance. ***P < 0.001.

We established a genome-edited mouse model by introducing the CRISPR-Cas9 system using single-stranded oligodeoxynucleotides into embryos obtained by *in vitro* fertilization via electroporation (Fig. 1). Sequencing was performed on tail DNA using two primer pairs: 5′-ATGGAGATGATCGACGTGGC-3′ (forward primer for detecting both wild-type *Fus* and *Fus* mutant), 5′-CTCTCCCTGCGATCCTGTCTGTG-3′ (reverse primer for detecting wild-type *Fus*) and 5′-CTCTCTCTCCGGTCCTGCCTATC-3′ (reverse primer for detecting *Fus* mutant). Wild-type C57BL/6 mice were purchased from the Jackson Laboratory, which were then directly inter-crossed with FUS genome-edited mice (F0). The Ethics Committee of Keio University approved all animal experiments, which were conducted according to the Animal Experimentation Guidelines of Keio University School of Medicine. The ARRIVE Reporting guidelines were followed for manuscript preparation. Mice were housed on ventilated racks in a specific pathogen-free barrier facility under a 12 h–12 h light–dark cycle. Mice were group housed with their littermates to a maximum of four mice per cage and were given free access to food and water. A total of 60 mice were used in this study.

### Evaluation of motor functions

#### Accelerating rotarod test

We previously acquired an accelerating rotarod apparatus (Neuroscience Inc.), which was used to analyse motor function as mice aged.^10^ Briefly, the mice underwent three trials per day for three consecutive days, and the maximum time it took to fall off the rotarod was recorded.

#### DigiGait analysis

We performed treadmill gait analysis using the DigiGait System (Mouse Specifics).^19^ The DigiGait System is a tool for assessing gait in mice. The treadmill belt of the DigiGait apparatus was constructed using thermoplastic polyurethane. During the gait analysis, we quantified various parameters, including hindlimb stride length, paw angle, stride duration, swing duration and cadence; forelimb stride length, paw angle, stride duration, swing duration and cadence; running speed and interlimb phase dispersion. The mice were subjected to treadmill locomotion at a rate of 7 cm/s, and their gaits were recorded as a video using the DigiGait software. Subsequently, the videos were analysed to ensure precise measurement of the aforementioned parameters.

### Immunoblot analysis

Mouse spinal cord was homogenized in cold lysis buffer. The buffer contained 50 mM NaCl, 10 mM Tris-HCl (pH 7.6), 30 mM sodium pyrophosphate, 20 mM glycerol 2-phosphate disodium salt hydrate, 1 mM EDTA, 1 mM EGTA, 1% Triton X-100, 0.05% sodium dodecyl sulphate and a protease inhibitor mixture (complete EDTA-free, Roche, 11836170001). Total protein concentrations in the supernatants were determined using the TaKaRa BCA Protein Assay Kit (Takara bio).

The proteins were then analysed via immunoblotting as follows. Protein separation and detection were performed using an automated capillary electrophoresis system, Wes (ProteinSimple), following the manufacturer’s protocol. Signals were detected with horseradish peroxidase-conjugated secondary antibodies and visualized using Compass for SW software (ProteinSimple).

The primary antibodies used in this study were as follows: anti-FUS (Bethyl, A304-293A, 1:50), anti-Lamin B1 (Abcam, #16048, 1:50), anti-Lamin A/C (Cell Signaling, #4777, 1:20) and β-actin as an internal loading control (Sigma, A1978, 1:50). Protein levels were determined by peak area using Compass for SW software (ProteinSimple).

### Immunohistochemical staining and imaging of mice

The basic procedure was performed as previously described.^20,21^ Mice were anaesthetized with an overdose of isoflurane (Pfizer). Brain and spinal cord tissues of mice were fixed using 4% paraformaldehyde in 1× PBS and embedded in O.C.T. compound (Sakura Finetek). Tissue sections (16 μm thickness) were obtained using a microtome (REM-710, Yamato Kohki Industrial Co., Ltd). Slides were stored at −30°C until use. After bringing the slides to room temperature, they were washed with 1× PBS for 3 min. Antigen retrieval was performed by autoclaving the slides in target retrieval solution (Dako, pH = 6.0) at 105°C for 5 min. After cooling, the slides were washed with 1× PBS for 5 min, permeabilized with 0.25% Triton X-100 diluted in 1× PBS for 10 min and washed three times, each for 5 min, with 0.1% Tween 20 in 1× PBS at room temperature in preparation for immunohistochemistry.

Immunohistochemistry was performed as previously described.^10,22,23^ Briefly, slides were blocked in blocking solution [1× PBS containing 1% bovine serum albumin (BSA), 1.5% goat or donkey serum and 0.1% Tween 20] for 2 h at room temperature. Subsequently, slides were incubated overnight at 4°C with primary antibodies diluted in a blocking solution.

The primary antibodies used in this study were as follows: anti-FUS (Bethyl, A304-293A, 1:500), anti-TDP-43 (Protein Tech, #12892-1-AP, 1:500), anti-G3BP (Protein Tech, #13057-2-AP, 1:1000), anti-Lamin B1 (Abcam, #16048, 1:500), anti-Lamin A/C (Cell Signaling, #4777, 1:100), anti-Nup62 (BD Bioscience, #610497, 1:200), anti-phospho-histone H2A.X (Ser139; Millipore, 05-636, 1:200), anti-choline acetyltransferase (Millipore, AB144P, 1:100), anti-mono- and poly-ubiquitinylated conjugate monoclonal antibody (FK2; ENZO, BML-PW8810-0100, 1:500) and anti-p62 (Millipore, MABC32, 1:500).

After incubation with primary antibodies, slides were washed three times, each for 5 min, with 0.1% Tween20 in 1× PBS, then incubated with secondary antibodies conjugated to Alexa Fluor 488, 555 or 647 (1:200, Thermo Fisher Scientific) diluted in blocking solution for 1 h at room temperature. Slides were washed three times, each for 5 min, with 0.1% Tween 20 in 1× PBS. Nuclei were stained with 4′,6-diamidino-2-phenylindole (DAPI). Finally, slides were coverslipped using a fluorescence mounting solution (Dako).

Immunofluorescence images were obtained using an LSM 780 laser scanning microscope (Zeiss) or Keyence BZ7000 microscope. Fluorescence intensity was determined via densitometry using ImageJ. All images were acquired using identical exposure times. Circularity was measured using ImageJ using the following formula: circularity = 4π(area) / (perimeter squared). The closer the circularity value to 1, the more circular the shape.

### Differentiation of hiPSC-derived lower motor neurons

Lower motor neuron (LMN) differentiation of hiPSCs and the neurite length analysis were performed as previously described.^24,25^ Briefly, LMNs were induced from hiPSCs using a chemically transitional embryoid-body-like state. The cells were infected with SeV-Lhx3 (LIM/homeobox protein 3)-Ngn2 (Neurogenin 2)-Isl1 (Islet-1) (ID Pharma) for RNA sequencing or with SeV-Lhx3-Ngn2-Isl1-EGFP (ID Pharma) for neurite length analysis. RNA samples were collected at 14 days of differentiation, and neurite outgrowth was assessed using the Biostation CT (Nikon) from Days 3 to 14 after initiating LMN induction.

The iPSC-LMNs used were as follows: 201B7 (Healthy-1)^26^; 414C2 (Healthy-2), WD39 (Healthy-3; from healthy donor)^27^; FALS-2e3 (H517D hetero-1)^18^; FALS-e48 (H517D hetero-2) [from patients with familial amyotrophic lateral sclerosis (FALS) and *FUS*^H517D/WT^]^18^; and FALS-Cre3 (H517D homo) (homozygous *FUS*^H517D/H517D^ mutation generated from healthy donor 409B2).^18^

### RNA sequencing analysis of hiPSC-derived lower motor neurons

Total RNA was extracted from hiPSC-LMNs and purified using RNeasy Mini kit (Qiagen) with DNase I treatment. Subsequently, the quantity and quality of RNA were confirmed using an Agilent TapeStation (Agilent Technologies). Complementary DNA was amplified using the SMART-Seq v4 Ultra Low Input RNA Kit for Sequencing (Takara Bio). A DNA library was then constructed using the Nextera XT DNA Library Prep Kit (Illumina). RNA sequencing was performed on a NovaSeq system (Illumina). All sequencing procedures were performed using Takara Bio. Fastp (version 0.23.2; all arguments were set to default) was used for quality control.^28^ Salmon [version 1.6; all arguments were set to default; gencode, release 42 (GRCh38.p13) was set as the reference genome] was used for read count quantification of sample data that passed quality control.^29^ The output files of Salmon were converted into a read count matrix using tximport (version 1.22.0).^30^ EBSeq was used for group comparisons.^31^ Gene Ontology (GO) enrichment [biological pathway (BP), cellular component (CC) and molecular function (MF)] and Kyoto Encyclopedia of Genes and Genomes (KEGG) pathway analyses were conducted using g:Profiler.^32-37^ The ‘Data sources’ option in g:Profiler was set to include ‘GO biological pathway’, ‘GO cellular component’, ‘GO molecular function’ and ‘KEGG’. The ‘term size’ option in g:Profiler was set to ‘5-350’ based on the recommendation of Reimand *et al*.^38^ STAR (version 2.7.10b) and arriba (version 2.3.0; all arguments were set to default) were used for fusion gene analysis.^39,40^

### Immunostaining and confocal imaging of hiPSC-derived lower motor neurons

On Day 14 of differentiation, iPSC-LMNs were plated in eight-well chamber slides (AGC Techno Glass Co., Ltd). At Day 14 of differentiation, iPSC-LMNs were fixed in 4% paraformaldehyde in 1× PBS, washed with 1× PBS three times, each for 5 min, permeabilized with 1× PBS containing 0.25% Triton X-100 for 15 min, blocked in blocking solution (2% normal goat serum diluted in 1× PBS) for 1 h, and incubated overnight at 4°C in primary antibodies diluted in blocking solution. The primary antibodies used were as follows: anti-FUS (Bethyl, A304-293A, 1:500), anti-Lamin B1 (Abcam, #16048, 1:500), anti-Lamin A/C (Cell Signaling, #4777, 1:100), anti-Nup62 (BD Bioscience, #610497, 1:200), anti-ISLET-1 (DSHB, 39.4D5, 1:1000), anti-HB9 (DSHB, 81.5C10, 1:150), anti-βIII-TUBULIN (Covance, MMS-435P, 1:2000) and anti-choline acetyltransferase (Millipore, AB144P, 1:100). After incubation with primary antibodies, slides were washed three times, each for 5 min, with 1× PBS, then incubated for 1 h at room temperature with secondary antibodies conjugated to Alexa Fluor 488, 555 or 647 (1:1000, Thermo Fisher Scientific) diluted in blocking solution. Slides were then washed three times, each for 5 min, with 1× PBS. The nuclei were stained with DAPI. Slides were coverslipped using a fluorescence mounting solution (Dako).

At Day 7 of differentiation into LMNs, lentivirus-CMV-NLS-tdTomato-NES (Shuttle-tdTomato, or S-tdTomato)^41,42^ was transduced at a multiplicity of infection (MOI) of 5, using the number of cells plated at Day 0, and incubated in 4 h with polybrene (4 μg/ml). Immunofluorescence images were obtained using an LSM 780 laser scanning microscope (Zeiss). The intensities of Lamin B1, Lamin A/C and Nup62 were quantified using ImageJ. All images were acquired using identical imaging parameters, such as laser power and gain.

### Immunohistochemical staining of human post-mortem spinal cord

Formalin-fixed, paraffin-embedded tissue sections (6-µm thick) were acquired from the Department of Pathology, Keio University School of Medicine (Tokyo, Japan) and the Department of Neurology, Tohoku University Graduate School of Medicine (Sendai, Japan). This study adhered to the principles of the Declaration of Helsinki and was approved by the Ethics Committee of the Keio University School of Medicine (No. 20080016 and No. 20160273) and the Tohoku University Graduate School of Medicine (No. 20221927). Supplementary Table 1 presents patient demographics.

Tissue sections were gradually rehydrated with xylene three times, each for 10 min, 100% ethanol twice for 30 s, 90% for 30 s, 70% for 30 s, 50% for 30 s, and finally rinsed three times with dH_2_O. Antigen retrieval was performed using an autoclave for 10 min at 120°C with target retrieval solution (Dako, pH = 6.0). After the slides were cooled, they were washed three times, each for 5 min, with 1 × PBS, permeabilized with 0.25% Triton X-100 diluted in 1× PBS for 10 min and washed three times, each for 5 min, in 1× PBS. Slides were incubated in 0.3% H_2_O_2_ for 1 h and blocked with 5% BSA at room temperature for 20 min. After blocking, slides were incubated overnight at 4°C with primary antibody diluted in 5% BSA. The primary antibodies used were as follows: anti-Lamin B1 (Abcam, #16048, 1:500) and anti-Nup62 (BD Bioscience, #610497, 1:200). After incubating with primary antibodies, slides were washed three times, each for 5 min, with 1× PBS, then incubated with the secondary antibody (biotinylated secondary antibody, 1:1000) diluted in 5% BSA at room temperature for 1 h. Slides were then washed three times, each for 5 min, in 1× PBS and stained with horseradish peroxidase/3,3′-diaminobenzidine tetrahydrochloride (DAB) using the Vectastain Elite ABC Kit (Vector Laboratories) until the slides reached the desired colour intensity. Slides were dehydrated using ethanol (50%, 70%, 90% and 100%) for 30 s each, and rinsed three times with xylene. Finally, slides were coverslipped using a mounting solution (Mount Quick). Immunohistochemical staining images were obtained using a Keyence BZ7000 microscope.

### Statistical analysis

Data analysis and visualization were performed in R version 4.2.3 using the following packages: ‘ggplot2’ (version 3.4.2), ‘corrplot’ (version 0.92), ‘multcomp’ (version 1.4.0) and ‘survival’ (version 3.5-5).^43-47^ Data collection and analyses were not performed blindly to the conditions of the experiment. *P*-values were calculated from two-sided tests, and statistical significance was set at *P* < 0.05.

## Results

### Generation of the genome-edited mice with H517D mutation in *FUS*

The proline–tyrosine NLS of FUS proteins, comprising three consensus components, including a basic charged sequence, a downstream conserved arginine and a proline–tyrosine sequence, is conserved across species (Fig. 1A). We used the CRISPR-Cas9-based genome editing approach to generate the *FUS*^WT/H517D^ genome-edited mice. The mouse position corresponding to human H517 is H509 (Fig. 1B). After genome editing, the altered sequence (H509 to D) was confirmed using direct sequencing (Fig. 1C). We analysed the levels of FUS protein in the spinal cord of *Fus*^WT/H509D^ mice using an anti-N-terminus FUS antibody. The total FUS levels were increased by ∼1.6-fold in the spinal cord of *Fus*^WT/H509D^ genome-edited mice, suggesting an altered protein turnover of the mutant protein (Fig. 1D and E). Therefore, we concluded a successful establishment of *Fus*^WT/H509D^ genome-edited mice.

### Progressive motor impairment in the *Fus*^WT/H509D^ genome-edited mice

**Figure 2:**
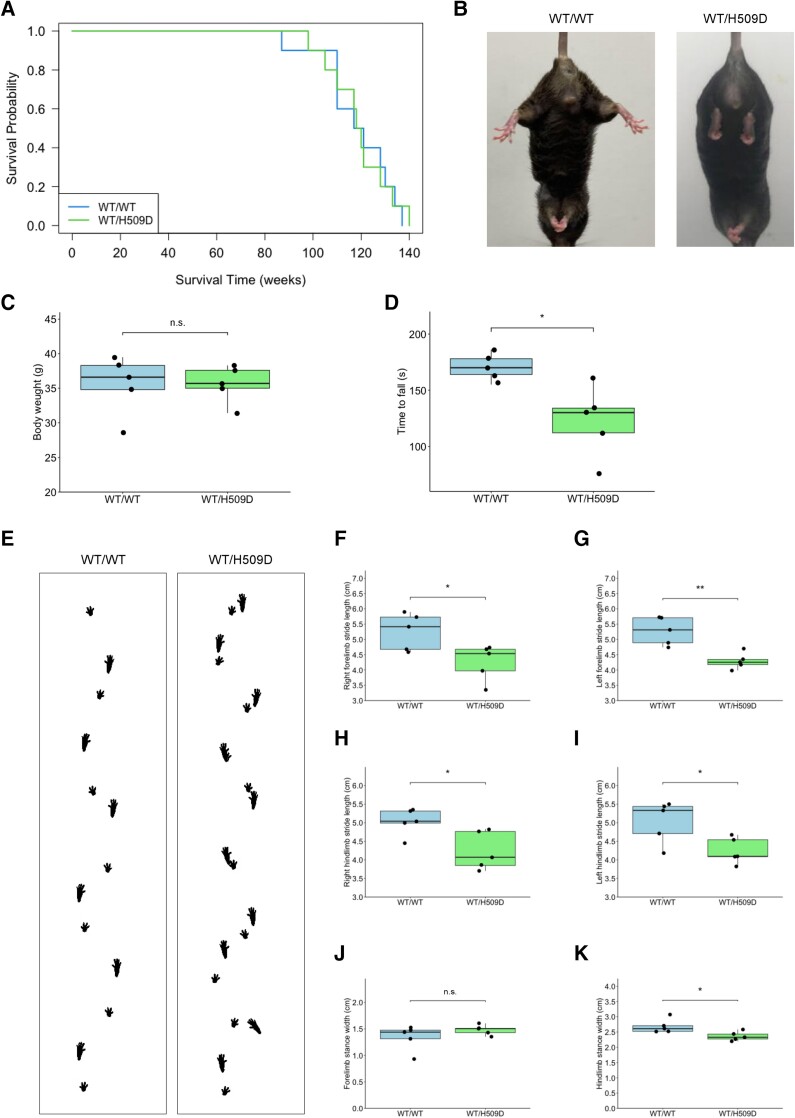
**Phenotypes of *Fus*^WT/H509D^ mice.** (**A**) Kaplan–Meier survival analysis of wild-type (WT) and *Fus*^WT/H509D^ mouse lines (*n* = 10, male = 5 and female = 5 for each group). Statistical significance was calculated using the log-rank test. No significant difference was observed in the survival curves between wild-type and *Fus*^WT/H509D^ mice. (**B**) Representative picture of an abnormal limb posture in *Fus*^WT/H509D^ mice. At 12 months, *Fus*^WT/H509D^ mice exhibited abnormal reflexes. (**C** and **D**) Body weight and accelerating rotarod test of wild-type and *Fus*^WT/H509D^ mice (25 months, *n* = 5 per genotype, male). No significant difference was observed in body weight between wild-type and *Fus*^WT/H509D^ mice (**C**); however, mutant mice exhibited motor deficits in the accelerating rotarod test (**D**). (**E**) Footprints of wild-type and *Fus*^WT/H509D^ mice at 25 months. (**F**–**K**) DigiGait analysis in wild-type and *Fus*^WT/H509D^ mice (25 months, *n* = 5 per genotype, male). Graphs represent quartiles (boxes), 50th percentile (centreline) and 1.5 times the interquartile range (whiskers). Student’s *t*-test was used to calculate statistical significance. *P < 0.05 and **P < 0.01. n.s. = no statistical significance.

During backcrossing with wild-type C57BL/6 mice, genome-edited mice were born at normal Mendelian ratios. Survival curves and body weight of wild-type and *Fus*^WT/H509D^ mice were not significantly different (Fig. 2A and C). *Fus*^WT/H509D^ mice showed an abnormal hindlimb reflex characterized by retraction of the hindlimbs upon lifting by the tail at 12 months (Fig. 2B).

Next, we used the accelerating rotarod to assess the motor functions of wild-type and *Fus*^WT/H509D^ mice at 18 and 25 months. The rotarod test showed motor impairment, which was prominent in the *Fus*^WT/H509D^ mice at 25 months (Fig. 2D), and did not differ until 18 months (Supplementary Fig. 1A). Gait abnormalities were also examined using a footprint test via the DigiGait System (Fig. 2E). At 18 months, the stride length of the forelimbs was significantly shorter in *Fus*^WT/H509D^ mice, but not in the hindlimbs (Supplementary Fig. 1B–E). Furthermore, at 25 months, the stride length of the forelimbs and hindlimbs and the stance width of hindlimbs were shorter in *Fus*^WT/H509D^ mice (Fig. 2F–K) than those in the wild-type mice. Collectively, although the survival of *Fus*^WT/H509D^ mice was unaffected, they exhibited progressive ALS-like motor impairment with ageing.

### Motor neuron loss and FUS mislocalization into the cytoplasm of *Fus*^WT/H509D^ mice

**Figure 3:**
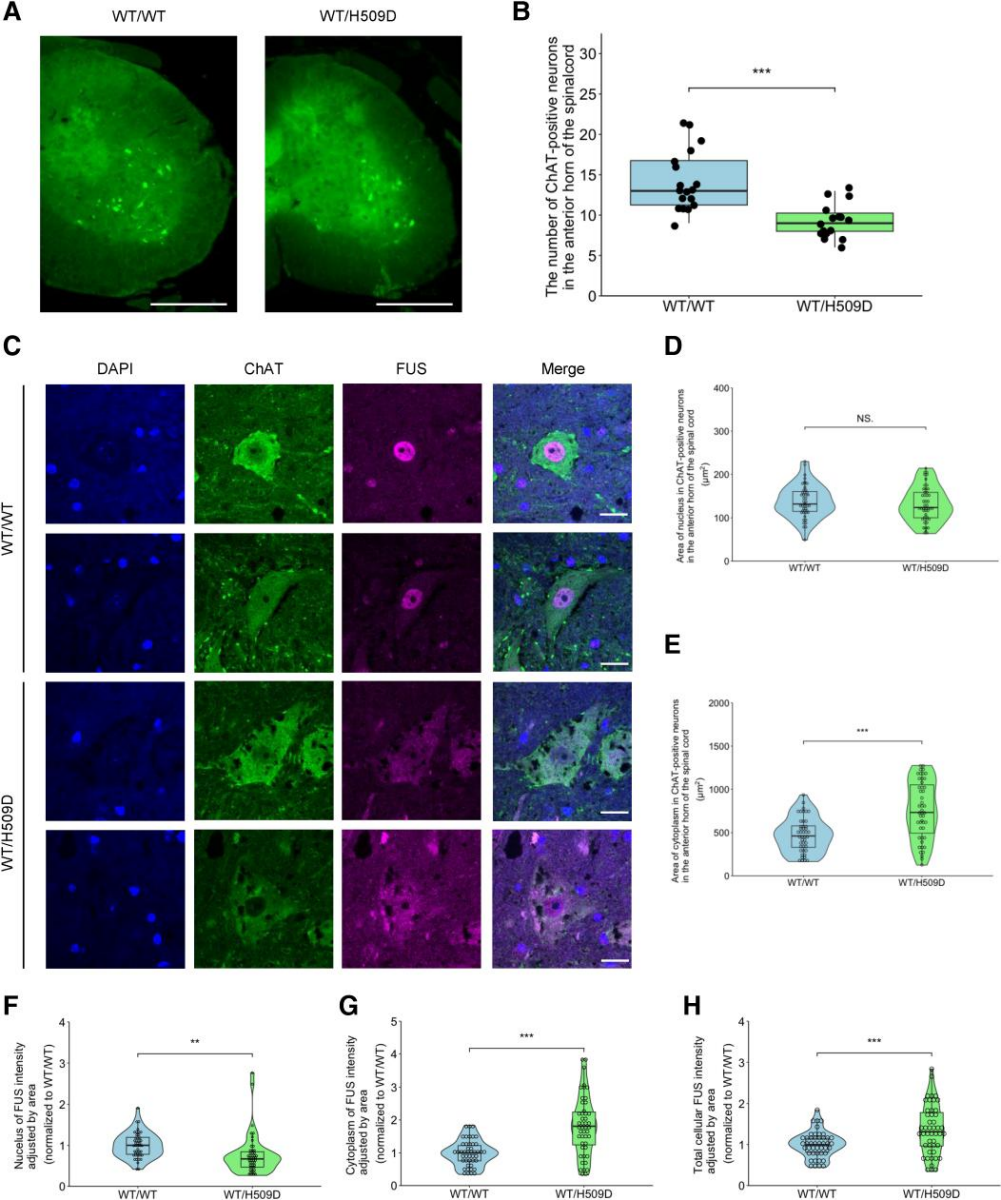
**Motor neuron loss and FUS mislocalization into the cytoplasm in the spinal cord of *Fus*^WT/H509D^ mice.** (**A**) Immunofluorescence images of the spinal cord with choline-acetyltransferase (ChAT) (green) staining in wild-type (WT) and *Fus*^WT/H509D^ mice at 18 months. Scale bars = 200 μm. (**B**) Boxplot showing the number of ChAT^+^ neurons in the anterior horn of the spinal cord in wild-type and *Fus*^WT/H509D^ mice (18 months, *n* = 3 per genotype, six sections per individual, male). Graphs represent quartiles (boxes), 50th percentile (centreline) and 1.5 times the interquartile range (whiskers). The Wilcoxon signed-rank test was used to calculate statistical significance. ***P < 0.001. (**C**) Immunofluorescence images of the spinal cord with ChAT (green) and FUS (magenta) staining in wild-type and *Fus*^WT/H509D^ mice at 18 months. Scale bars = 20 μm. (**D** and **E**) Box plots showing quantification of the area of the nucleus (**D**) and cytoplasm (**E**) in ChAT^+^ neurons in the anterior horn of the spinal cord (18 months, *n* = 3 per genotype, 46 cells wild-type; 48 cells *Fus*^WT/H509D^, male). ***P < 0.001. (**F**–**H**) Box plots showing quantification in the nucleus of FUS (**F**), in the cytoplasm of FUS (**G**) and total cellular FUS (**H**) in ChAT^+^ neurons in the anterior horn (18 months, *n* = 3 per genotype, 46 cells wild-type; 48 cells *Fus*^WT/H509D^, male). **P < 0.01 and ***P < 0.001.

To characterize MN degeneration, we counted the number of choline-acetyltransferase (ChAT)-positive MNs in the anterior horn of the spinal cord. Immunohistochemistry revealed significant MN loss in *Fus*^WT/H509D^ mice at 18 months (Fig. 3A and B). We evaluated the areas of the nucleus and cytoplasm and showed that nucleus did not change, but the cytoplasm expanded in the ChAT^+^ neurons in *Fus*^WT/H509D^ mice at 18 months (Fig. 3C–E).

The hallmark pathology of ALS with *FUS* mutation is cytoplasmic mislocalization and aggregation or inclusion of FUS protein in the MNs.^48^ To assess FUS localization, we analysed the FUS intensity ratio of the nucleus, cytoplasm and nucleus/cytoplasm (N/C) adjusted by area. Immunohistochemistry with FUS in the spinal cord showed FUS mislocalization to the cytoplasm and increased total cellular FUS in the ChAT^+^ neurons in *Fus*^WT/H509D^ mice at 18 months (Fig. 3C and F–H), consistent with western blot analysis (Fig. 1D and E).

We also did not detect p62/ubiquitin-positive inclusions in spinal MNs (Supplementary Fig. 2A and B). Therefore, these results suggest that FUS mislocalization leads to motor phenotypes without FUS inclusions.

In contrast, there was no obvious increased intensity of IBA1 (ionized calcium-binding adapter molecule 1) and GFAP (glial fibrillary acidic protein) in spinal cord of *Fus*^WT/H509D^ mice at 25 months, indicating that glial activation is not crucial for the pathomechanism in this murine model. (Supplementary Fig. 3A–C).

Increasing evidence suggests that ALS-associated mutations in TDP-43 and FUS lead to the formation of aberrant stress granules, the normal cellular defence mechanism against stress conditions via RNA quality control, *in vitro* and *in vivo*. In wild-type mice, G3BP1, a major component of stress granules, uniformly stained the cytoplasm in MNs. In contrast, in *Fus*^WT/H509D^ mice, staining patterns in most MNs showed drastically changed foci, with no uniform staining in the cytoplasm for G3BP1 (Supplementary Fig. 4A–C). TDP-43, an ALS-linked RNA-binding protein, also showed an abnormal cytoplasmic distribution (Supplementary Fig. 5A–C).

### Disruption of the nuclear lamina and nucleoporins in *Fus*^WT/H509D^ mice

As a next step, we investigated the phenotypes of the nuclear lamina and nucleoporins in *Fus*^WT/H509D^ mice in detail. Previous reports have shown that abnormal staining of the nuclear structural protein Lamin B1 was observed in cells derived from TDP-43 mutant patients.^49^ However, this abnormal staining was not observed in the induced neurons derived from *C9orf72* mutation carriers^50^ or in post-mortem samples of the motor cortex from *C9orf72* patients.^51^ Consequently, the morphology of the nuclear lamina in ALS remains a subject of controversy. Earlier studies on the nuclear pore complex (NPC) have revealed disrupted nucleoporins in *SOD1*-G93A transgenic mice^52^ and in post-mortem spinal cord samples from patients with sporadic ALS^53,54^ and *FUS*-ALS.^55^ Here, we evaluated the morphology of the nuclear lamina and nucleoporins of LMNs of *Fus*^WT/H509D^ mice.

**Figure 4:**
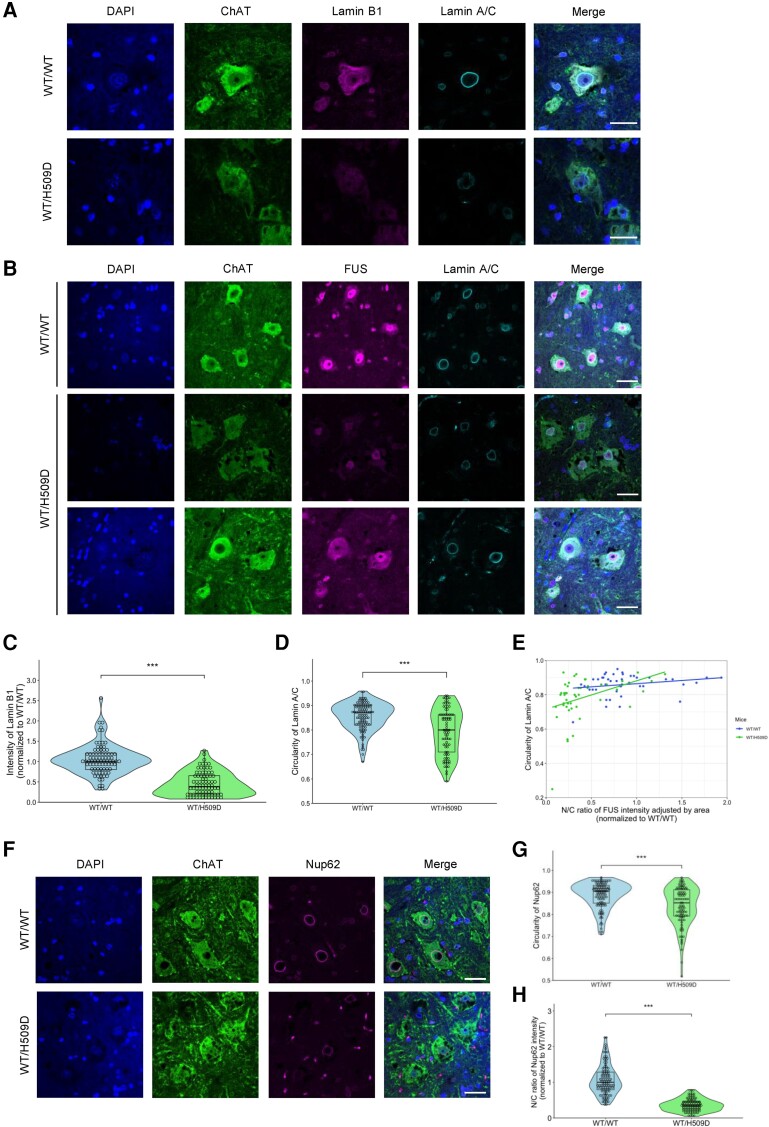
**Disruption of the nuclear Lamina B1 and Lamin A/C and nucleoporins in *Fus*^WT/H509D^ mice.** (**A**) Immunofluorescence images of the spinal cord with anti-ChAT (green), Lamin B1 (magenta) and Lamin A/C (cyan) antibodies in wild-type (WT) and *Fus*^WT/H509D^ mice at 18 months. Images are representative of *n* = 3 experiments. Scale bars = 20 μm. (**B**) Immunofluorescence images of the spinal cord with anti-ChAT (green), FUS (magenta) and Lamin A/C (cyan) antibodies in wild-type and *Fus*^WT/H509D^ mice at 18 months. Images are representative of *n* = 3 experiments. Scale bars = 20 μm. (**C**) Violin plot showing the quantification of Lamin B1 intensity in ChAT^+^ neurons in the anterior horn of the spinal cord in wild-type and *Fus*^WT/H509D^ mice (18 months, *n* = 3 per genotype, 84 cells wild-type, 81 cells *Fus*^WT/H509D^, male). Graphs represent quartiles (boxes), 50th percentile (centreline) and 1.5 times the interquartile range (whiskers). Student’s *t*-test was used to calculate statistical significance. ***P < 0.001. (**D**) Violin plot showing the quantification of nuclear lamina circularity in ChAT^+^ neurons in the anterior horn in wild-type and *Fus*^WT/H509D^ mice (18 months, *n* = 3 per genotype, 103 cells wild-type, 86 cells *Fus*^WT/H509D^, male). (**E**) Scatter plot showing circularity of Lamin A/C and nucleus/cytoplasm (N/C) ratio of FUS adjusted by area in ChAT^+^ neurons in the anterior horn (18 months, *n* = 3 per genotype, 46 cells wild-type, 48 cells *Fus*^WT/H509D^, male). Simple linear regression slope. ***P < 0.001. (**F**) Immunofluorescence images of the spinal cord with ChAT (green) and Nup62 (magenta) staining in wild-type and *Fus*^WT/H509D^ mice at 18 months. Scale bars = 20 μm. (**G** and **H**) Violin plots showing the quantification of nucleoporin (Nup62) circularity (**G**) and intensity ratio (**H**) in ChAT^+^ neurons in the anterior horn (18 months, *n* = 3 per genotype, 110 cells wild-type, 97 cells *Fus*^WT/H509D^, male). ***P < 0.001. ChAT = choline acetyltransferase.

To accomplish this, we conducted immunohistochemical analysis with Lamin B1 and Lamin A/C in the spinal cord. As shown in Fig. 4A and B, disruption of the nuclear lamina was evident in the spinal cord of *Fus*^WT/H509D^ mice. Quantification using ImageJ, an image analyser, showed a significant decrease of the intensity of Lamin B1 and circularity of Lamin A/C (Fig. 4C and D).

We analysed the levels of Lamin B1 and Lamin A/C protein in the spinal cord of *Fus*^WT/H509D^ mice quantitatively, by western blot. Although the total Lamin B1 level was not different between wild-type and *Fus*^WT/H509D^ genome-edited mice (Supplementary Fig. 6A and B), the ratio of modified Lamin B1 (upper and lower bands) was significantly different, showing aberrant post-translational modifications of Lamin B1 between wild-type and *Fus*^WT/H509D^ genome-edited mice (Supplementary Fig. 6C). The total Lamin A/C level, which includes alternative splicing products of the *Lmna* gene, was not different (Supplementary Fig. 6D and E), but the ratio of Lamin A to Lamin C was changed in *Fus*^WT/H509D^ genome-edited mice (Supplementary Fig. 6F). Together, bulk samples also showed abnormal levels of the lamin family.

We wanted to know whether the FUS mislocalization or the disturbance of nuclear lamina and nucleoporins occurred first. To answer this question, we stained Lamin A/C and FUS simultaneously and made a scatter plot (Fig. 4B and E). The localization of FUS was altered in ALS model mice despite no disruption to Lamin A/C. This finding suggests that FUS mislocalization might occur before nuclear membrane damage in *FUS*-ALS. Immunostaining of the main component of the NPC, Nup62, also exhibited disrupted and reduced staining patterns (Fig. 4F–H). Evaluation of the intensity ratio of N/C also revealed reduced Nup62 on the nuclear lamina in the spinal cord of *Fus*^WT/H509D^ mice at 18 months.

Furthermore, many lines of evidence show that FUS proteins play an important role in the repair of DNA damage,^12,56^ and lamina dysfunction and nuclear envelop ruptures are linked to DNA damage.^57^ We performed immunohistochemistry with γH2AX (phosphorylated H2AX), an established definitive marker of DNA damage, in the spinal cord to assess DNA damage. As shown in Supplementary Fig. 7A and B, the percentage of MNs with γH2AX foci increased significantly in *Fus*^WT/H509D^ mice, indicating that the expression of mutant FUS induces DNA damage in spinal LMNs.

Collectively, our mice showed mild motor deficits, with steadily progressing disruption of the nuclear lamina and nucleoporins and DNA damage in the MNs.

### Human iPSC-derived lower motor neurons (*FUS-*H517D) exhibit reduced expression of the nuclear lamina and nucleoporins

**Figure 5:**
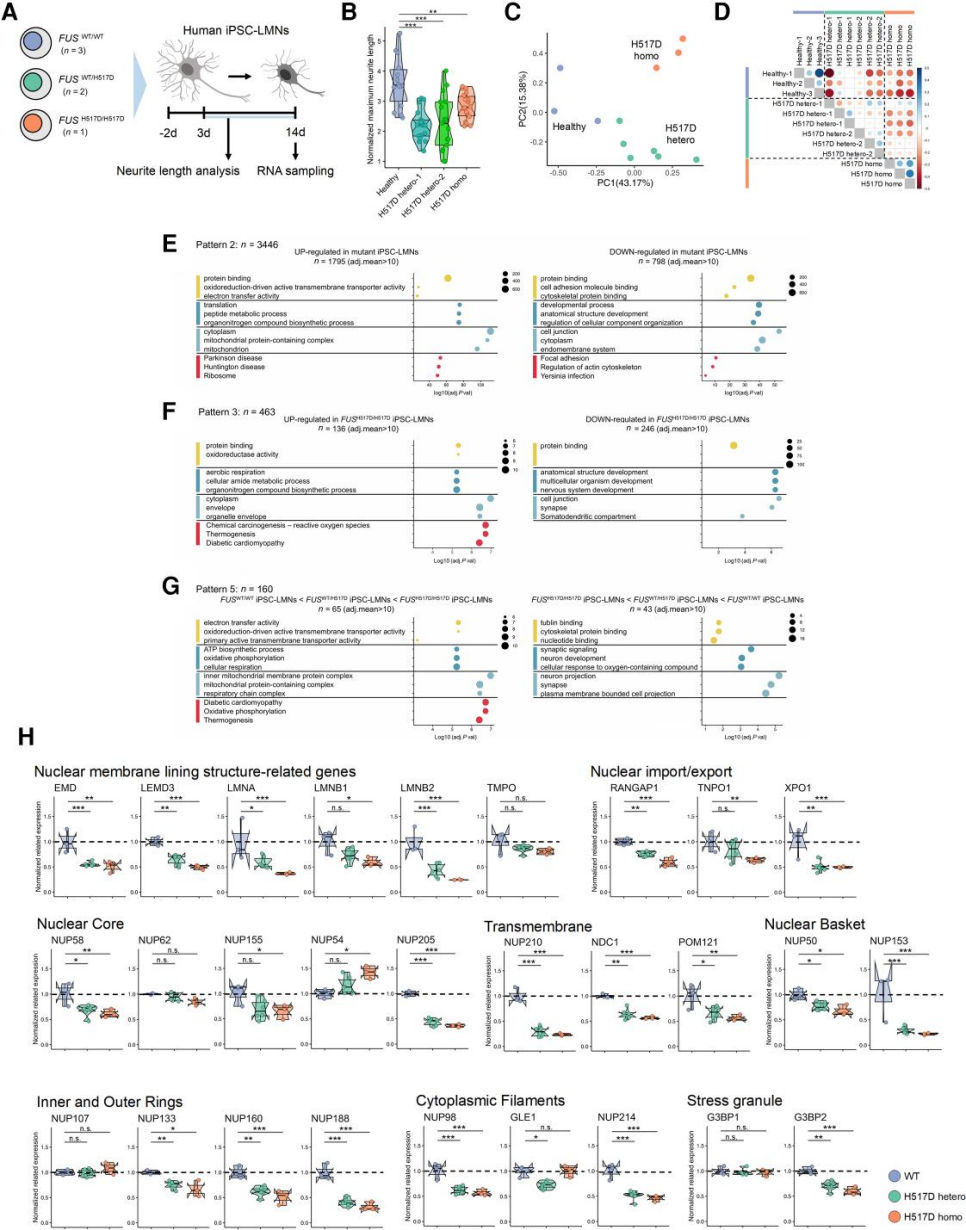
**RNA sequencing analyses revealed decreased expression levels of the nuclear lamina and nucleoporins in human induced pluripotent stem cell-derived lower motor neurons with *FUS*-H517D.** (**A**) Schematic diagram illustrating the generation of induced pluripotent stem cell-derived motor neurons (iPSC-MNs) from healthy donor-derived iPSCs (*n* = 3), patients with familial amyotrophic lateral sclerosis and *FUS*^WT/H517D^-derived iPSCs (*n* = 2), and iPSCs with the homozygous *FUS*^H517D/H517D^ mutation derived from healthy donor iPSCs (*n* = 1). After 2 days of incubation, neurite length was measured from Days 3 to 14, and RNA was collected on Day 14 of incubation. (**B**) The normalized maximum neurite length in iPSC-LMNs. Graphs represent quartiles (boxes), 50th percentile (centreline) and 1.5 times the interquartile range (whiskers). Dunnett’s test was used to calculate statistical significance. **P < 0.01 and ***P < 0.001. (**C** and **D**) Principal component (PC) analysis results for transcriptomes in Healthy-, H517D hetero- and H517D homo-LMNs (**C**) and Pearson correlation analysis (**D**) (purple, Healthy; green, H517D hetero-1 or -2; orange, H517D homo). (**E**–**G**) Results of gene expression analysis in Healthy-, H517D hetero-1- and 2- and H517D homo-LMNs. Significant differences in expression levels were observed between groups of different classes (alphabets) (the size of the expression levels was not considered). Results of functional analysis of gene group with significantly increased (**E**, *left*) or decreased (**E**, *right*) expression in H517D hetero- and H517D homo-LMNs compared with Healthy-LMNs, gene group with significantly increased (**F**, *left*) or decreased (**F**, *right*) expression in H517D homo-LMNs compared with H517D hetero- and Healthy-LMNs, and gene group whose expression was upregulated (**G**, *left*) or downregulated (**G**, *right*) in Healthy-, H517D hetero- and H517D homo-LMNs, in that order. The top three terms for each biological pathway, cellular component, molecular function and KEGG pathway are listed. Balloon size represents the number of genes belonging to each term. (**H**) Comparison of expression levels of the representative nuclear lamina, nuclear pore complex (NPC; scaffold, core and peripheral) and other interested genes. The vertical axis represents the relative expression of normalized count data to Healthy-LMNs (Benjamini–Hochberg method). *P < 0.05, **P < 0.01 and ***P < 0.001. n.s. = no statistical significance.

To explore in detail the effects of *FUS*^H517D^ mutation in human MNs, we obtained hiPSC-LMNs from two patients with FALS and *FUS*^WT/H517D^ mutation (H517D hetero-1, -2), three healthy participants (Healthy) and homozygous *FUS*^H517D/H517D^ mutation (H517D homo) generated in a previous study,^18^ and the *in vitro* data were compared. We used the rapid induction method to generate LMNs from hiPSCs, measured neurite length transition from Days 3 to 14, and collected total RNA for RNA sequencing on Day 14 (Fig. 5A).

We compared the normalized neurite length transition between hiPSC-LMNs generated from Healthy, H517D hetero-1, -2 and H517D homo (Fig. 5B). The normalized maximum neurite length of H517D hetero-LMNs and H517D homo-LMNs was significantly shorter than that of Healthy-LMNs.

In addition, RNA sequencing was performed on RNAs extracted from iPSC-LMNs 14 days after differentiation. Principal component analysis of the transcriptomes showed that the three groups (Healthy-LMNs, H517D hetero-LMNs and H517D homo-LMNs) were differentially distributed (Fig. 5C and Supplementary material). Moreover, Pearson correlation analysis of the transcriptomes in each sample suggested that Healthy-LMNs and H517D homo-LMNs differed significantly in nature, whereas H517D hetero-LMNs showed a distribution intermediate between the two (Fig. 5D). We also performed gene expression analysis in the three groups, including Healthy-LMNs, H517D hetero-LMNs and H517D homo-LMNs. The comparative expression analysis was performed using an empirical Bayesian model, and the gene expression patterns were classified into five patterns (Supplementary Table 2).

Functional analysis was performed for the gene group whose expression was significantly altered in H517D hetero-LMNs and H517D homo-LMNs (H517D mutant-LMNs) compared with Healthy-LMNs to examine the effect of *FUS*^H517D^ mutation (Fig. 5E, Pattern 2). The genes with upregulated expression in H517D mutant-LMNs compared with Healthy-LMNs were involved in translation and mitochondrial electron transport (Fig. 5E, left). In contrast, the genes with downregulated expression were involved in focal adhesion, cytoskeletal regulation and cellular component organization regulation.

Next, to examine the specific changes in the *FUS*^H517D/H517D^ mutation, we performed functional analysis on the gene group with significantly variable expression levels in H517D homo-LMNs compared with Healthy-LMNs and H517D hetero-LMNs (Fig. 5F, Pattern 3). The genes with upregulated expression in H517D homo-LMNs compared with H517D hetero-LMNs and Healthy-LMNs participated in redox reactions and aerobic respiration. In contrast, the genes with downregulated expression in H517D homo-LMNs compared with H517D hetero-LMNs and Healthy-LMNs participated in synaptogenesis. The genes that were significantly upregulated in Healthy-LMNs, H517D hetero-LMNs and H517D homo-LMNs, in that order (Fig. 5G, Pattern 5), were engaged in the electron transport system, ATP synthesis and oxidative phosphorylation. In contrast, the genes whose expression levels were downregulated participated in synaptic signalling.

Given the evidence of disruption of the nuclear lamina and nucleoporins in mice, we compared the expression of the representative nuclear membrane lining structure-related genes, nuclear import or export genes and nucleoporin genes. We discovered that the expression levels of most genes decreased significantly in H517D mutant-LMNs (Fig. 5H). Thus, our findings suggest that *FUS*^H517D^ mutation induces a decrease in the expression levels of major genes related to nuclear membrane lining structure, nucleocytoplasmic transport and nucleoporins.

Gene fusions are caused by double-stranded DNA breakages followed by a DNA repair error.^58^ Based on the increased γH2AX foci in *Fus*^WT/H509D^ mice (Supplementary Fig.7), the number of gene fusions was also examined using a gene fusion detection algorithm from RNA sequencing data of hiPSC-LMNs. In comparison to Healthy, H517D hetero showed a tendency to increase, and H517D homo showed a significantly increase in the number of fusion genes without any specific gene fusions (Supplementary Fig. 8 and Supplementary material), indicating genetic instability in H517D mutant-LMNs.

### Disruption of the nuclear membrane and nucleocytoplasmic transport in hiPSC-LMNs (*FUS*-H517D)

**Figure 6:**
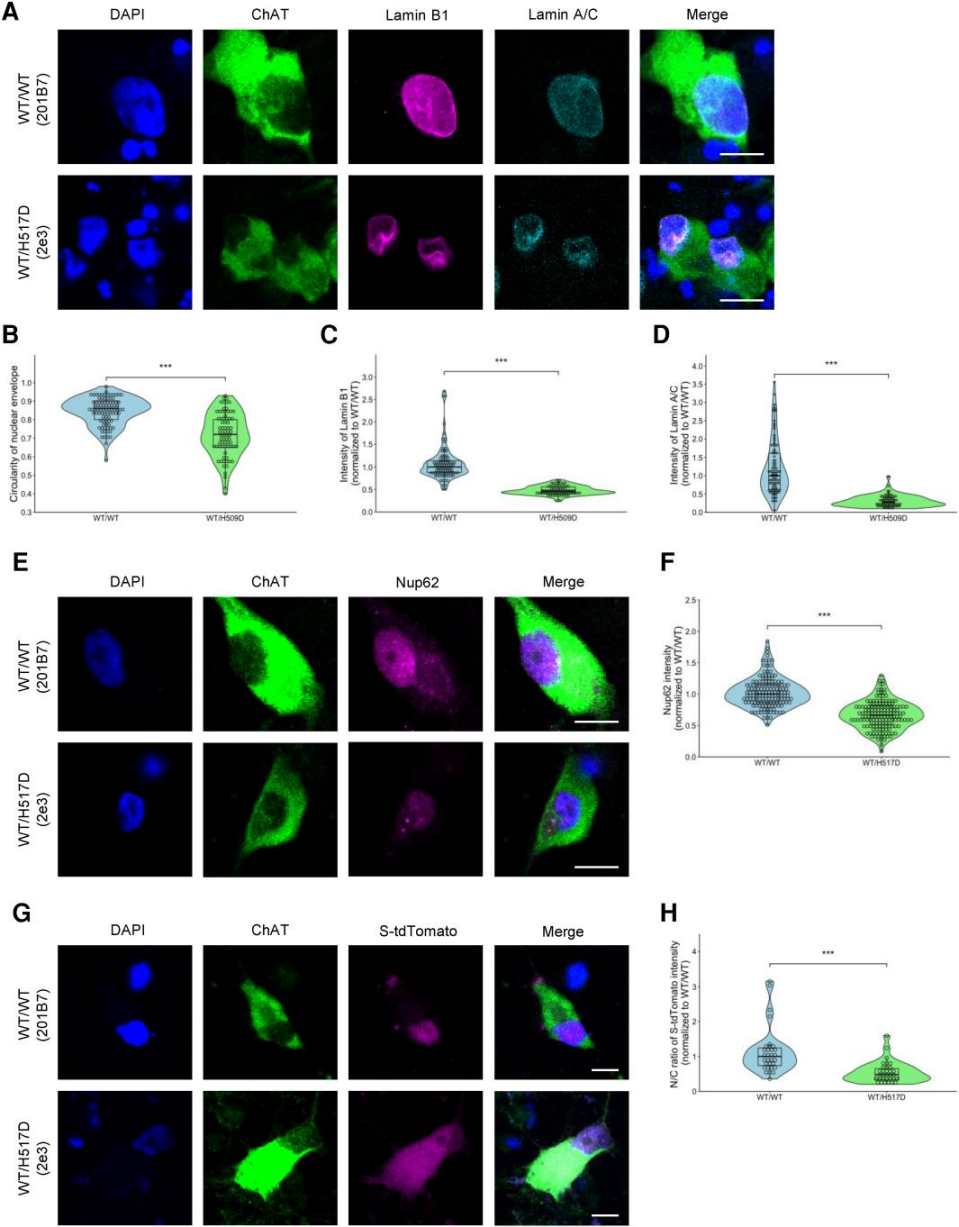
**Disruption of the nuclear lamina, nucleoporins and nucleocytoplasmic transport in lower motor neurons differentiated from human induced pluripotent stem cell-derived lower motor neurons with *FUS*-H517D**. (**A**) Immunofluorescence images of human induced pluripotent stem cell-derived lower motor neurons (hiPSC-LMNs) with anti-ChAT (green), Lamin B1 (magenta) and Lamin A/C (cyan) antibodies in Healthy-LMNs (201B7) and H517D hetero-1-LMNs on Day 14 of differentiation. Images are representative of *n* = 3 independent experiments. Scale bars = 10 μm. (**B**) Violin plot showing quantification of the nuclear lamina circularity (Lamin B1) in ChAT^+^ neurons (*n* = 3 for each, 90 cells Healthy, 73 cells H517D hetero). Graphs represent quartiles (boxes), 50th percentile (centreline) and 1.5 times the interquartile range (whiskers). Student’s *t*-test was used to calculate statistical significance. ***P < 0.001. (**C** and **D**) Violin plots showing quantification of the intensity of Lamin B1 and Lamin A/C in ChAT^+^ neurons (*n* = 3 for each, 90 cells Healthy, 73 cells H517D hetero). ***P < 0.001. (**E**) Immunofluorescence images of iPSC-MNs with anti-ChAT (green) and Nup62 (magenta) antibodies in Healthy- and H517D hetero-1-MNs on Day 14 of differentiation. Scale bars = 10 μm. (**F**) Quantification nucleus/cytoplasm (N/C) ratio of Nup62 intensity in ChAT^+^ neurons (*n* = 3 per each, 150 cells Healthy, 155 cells H517D hetero). ***P < 0.001. (**G**) Immunofluorescence images of iPSC-MNs with anti-ChAT (green) antibody and S-tdTomato (magenta) in Healthy- and H517D hetero-1-MNs on Day 14 of differentiation. Scale bars = 10 μm. (**H**) Quantification of nucleus/cytoplasm (N/C) ratio of S-tdTomato intensity in ChAT^+^ neurons (*n* = 3 per each, 31 cells Healthy, 32 cells H517D hetero). ***P < 0.001.

We used the *FUS*^WT/H517D^ mutation (H517D hetero-1 or FALS-2e3) as our mouse model to investigate the potential impact on the nuclear lamina and nucleoporins. For this purpose, immunocytochemistry was conducted using Lamin B1, Lamin A/C and Nup62 antibodies on hiPSC-derived ChAT^+^ MNs at Day 14 of differentiation. We evaluated the circularity of the nuclear lamina and the intensity of Lamin B1, Lamin A/C and Nup62 to examine the disruption of the nuclear lamina and nucleoporins. As shown in Fig. 6A, an aberrant nuclear lamina was evident in *FUS*^WT/H517D^-mutant LMNs. We quantified the circularity and intensity of nuclear lamina using ImageJ and reveal disruption of the nuclear lamina in Fig. 6B–D. Next, we also analysed the intensity of Nup62 and show the decreased expression of Nup62 in Fig. 6E and F. Thus, these findings are consistent with our results and previous reports, suggesting disruption of the nuclear lamina and nucleoporins attributable to *FUS*^H517D^ mutation. Furthermore, we used tdTomato proteins tagged with nuclear localization signals (NLS) and nuclear export signals (NES) (S-tdTomato) to analyse the function of nucleocytoplasmic transport, which shuttles between the cytoplasm and the nucleus.^41,42^ In healthy LMNs, S-tdTomato is localized primarily to the nucleus, but in *FUS*^WT/H517D^-mutant LMNs, S-tdTomato is distributed to the cytoplasm (Fig. 6G and H). Thus, our results suggest that the *FUS*^H517D^ mutation leads to dysfunction of nucleocytoplasmic transport associated with disruption of the nuclear lamina and nucleoporins.

### Disruption of the nuclear lamina and nucleoporins in sporadic and familial ALS human post-mortem spinal cord

**Figure 7:**
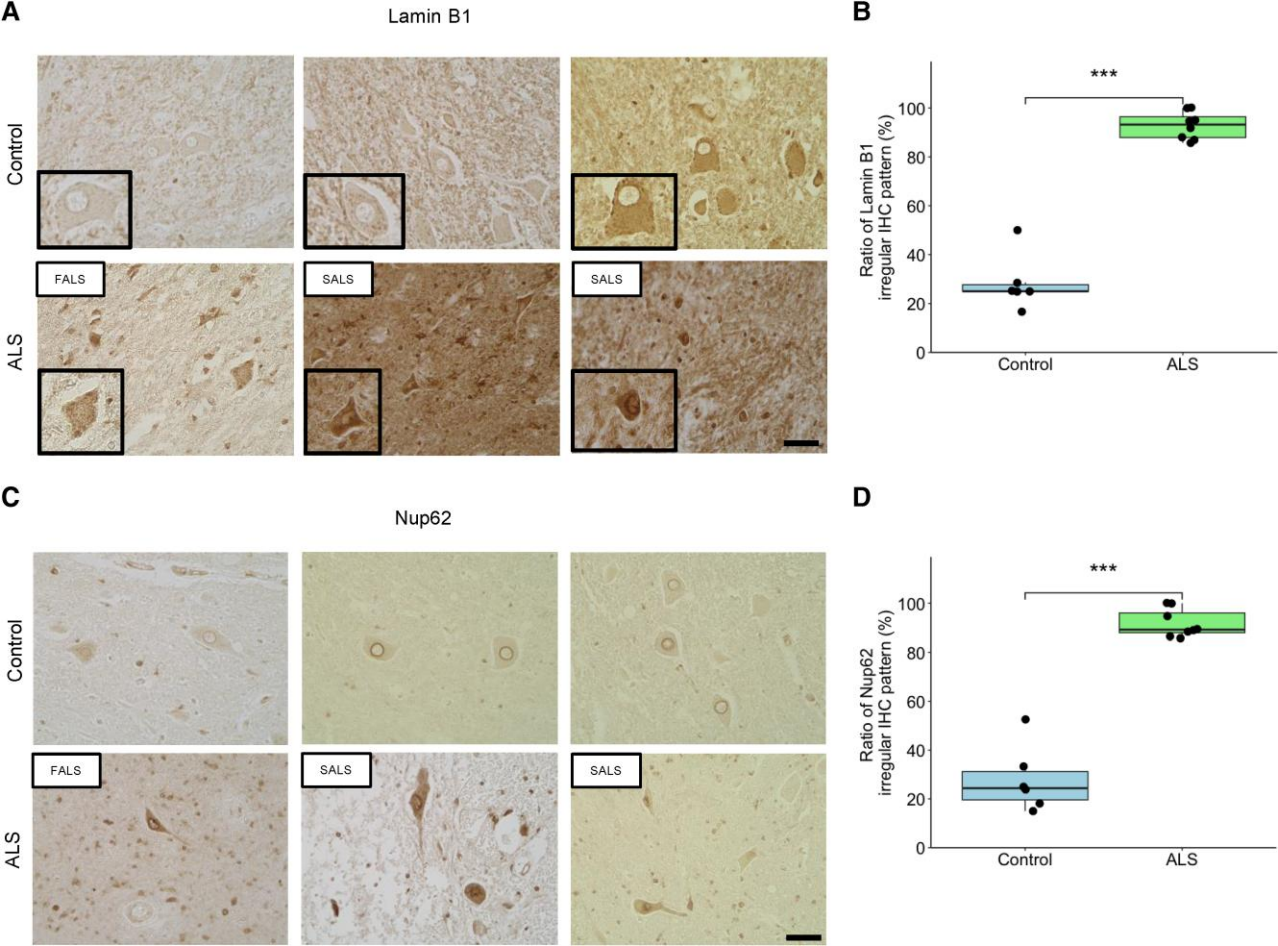
**Disruption of the nuclear lamina and nucleoporins in sporadic amyotrophic lateral sclerosis and familial amyotrophic lateral sclerosis of human post-mortem spinal cord.** (**A**) Immunohistochemistry of Lamin B1 in human post-mortem spinal cord. Images are representative of *n* = 6–8 staining experiments. Scale bar = 50 μm. (**B**) The ratio of Lamin B1 irregular immunohistochemistry patterns in control and amyotrophic lateral sclerosis (ALS) of human post-mortem spinal cord. Graphs represent quartiles (boxes), 50th percentile (centreline) and 1.5 times the interquartile range (whiskers). The Mann–Whitney U-test was used to calculate statistical significance. ***P < 0.001. (**C**) Immunohistochemistry of Nup62 in human post-mortem spinal cord. Images are representative of *n* = 6–8 staining experiments. Scale bar = 50 μm. (**D**) The ratio of Nup62 irregular immunohistochemistry patterns in control and ALS of human post-mortem spinal cord. Mann–Whitney U-test was used to calculate statistical significance. ***P < 0.001. FALS = familial amyotrophic lateral sclerosis; SALS = sporadic amyotrophic lateral sclerosis.

Finally, we investigated the disruption of nuclear lamina and nucleoporins in the human post-mortem spinal cord from sporadic amyotrophic lateral sclerosis (SALS) and *FUS*-FALS. We evaluated R521L, which has a mutation in the NLS, because *FUS*-H517D of human post-mortem tissue is unavailable. In the control cases, the disruption of Lamin B1 and Nup62 staining were observed in some cells; however, most cells had preserved morphology. In the ALS cases, although the number of LMNs was reduced, the remaining cells showed disruption of Lamin B1 and Nup62 staining (Fig. 7). The cells that had preserved morphology also showed an aberrant Lamin B1 and Nup62 staining pattern. Collectively, the disruption of the nuclear lamina and nucleoporins is a crucial pathology in *FUS*-ALS cases and sporadic ALS, suggesting that this pathomechanism is common across various forms of ALS.

## Discussion

In the present study, we successfully established a new ALS animal model, *Fus*^WT/H509D^ mice, using CRISPR-Cas9, and provided multiple lines of evidence showing that the disruption of the nuclear lamina and nucleoporins is crucial to the pathomechanisms of *FUS*-ALS. There was an increased FUS protein level in the spinal cords of genome-edited mice, possibly attributable to changes in structure and intracellular distribution caused by the large charge change, *FUS*-H517D mutation, which alters the degradation process of mutant FUS. This mouse strain showed progressive motor impairment with ageing (DigiGait analysis and rotarod test), which was linked to neuronal loss in the spinal cord without FUS inclusions in the cytoplasm (Fig. 3). The pedigree of *FUS*-H517D was reported from Japan, and its clinical features were upper limb-predominant onset at ∼40 years of age.^59^ In our mice, DigiGait analysis showed that the stride length of the forelimbs was shorter than that of the hindlimbs at 18 months (Fig. 2E–K), recapitulating the predominant clinical features of the upper limb onset in this rare mutation.

In genetically modified ALS animal models, which maintain physiological expression levels of FUS and TDP-43, recapturing prominent ALS motor phenotypes in heterozygous mutant animals is challenging. Homozygous genetically modified mice are frequently analysed to elicit prominent phenotypes; however, the effect of function loss in target genes deviates from the clinical significance of having only heterozygous patients with ALS.

In the TDP-43 knock-in mouse model of ALS, many studies did not reveal prominent motor deficits in heterozygous mice (including Q331K, M337V and G298S).^60-62^ Huang *et al*.^63^ reported that *TDP-43*^WT/N390D^ mice, but not *TDP-43*^WT/A315T^ mice, develop ALS pathologies at the molecular, cellular and behavioural (rotarod) levels, indicating mutation owing to differential effects in the mouse and human genetic backgrounds. Scekic-Zahirovic *et al*.^64^ reported a conditional knock-in heterozygous mouse model (*FUS*-ΔNLS mice), in which the NLS of FUS was deleted. They revealed delayed MN degeneration, neuropathological changes and mild motor impairment (shorter hanging time in an inverted grid test and irregular walking pattern) at 10 months.^64^ In humanized *FUS*-ALS knock-in mice with frame-shifted C-terminus mutation, skipping exon 14 in heterozygous animals shows hindlimb motor deficits at 16–18 months and motor degeneration. Zhang *et al*.^65^ established a genome-edited *FUS*-ALS point mutation, R521C, knock-in mouse model, which carries subtle impaired motor ability (including decreased travel distance in the open field test and stay time on the rotarod) at 7 months, late-onset MN loss, and stress granule misprocessing after sodium arsenite treatment. Our mice also exhibited delayed mild motor deficits (Fig. 2), indicating that it might take time for the physiological expression levels of mutant FUS to exert motor impairment of age-dependent neurodegeneration; however, a single copy of ALS point mutation of FUS can lead to ALS degeneration and phenotype on mouse genetic backgrounds.

Another new finding is that prominent disruption of the nuclear lamina and nucleoporins occur with mild motor deficits in this mouse strain (Fig. 4A–E). The patient-derived hiPSC-MNs with *FUS*^H517D^ also show remarkable disruption of the nuclear lamina at 14 days after motor differentiation (Figs 5I and 6A–D and Supplementary Fig. 6). These findings are consistent with previous findings on patient-derived hiPSC-MNs with *FUS*^M511Nfs^ mutation^49^ and human post-mortem spinal cord with *FUS*-P525L mutation,^54^ indicating common pathology in *FUS*-ALS. Therefore, disruption of the nuclear lamina and nucleoporins might be a crucial pathomechanism, possibly from the early phase of *FUS*-ALS.

However, the molecular mechanism of the nuclear lamina and nucleoporin disruption caused by *FUS* mutation is unclear. Recent studies have revealed that pathological TDP-43 and FUS protein and nucleoporins co-aggregate in the cytoplasm, thereby resulting in mislocalization of nucleoporins.^66,67^ Lin *et al*.^67^ reported that aberrant mutant FUS–Nup62 interactions can form an amorphous assembly reminiscent of insoluble oligomers based on the patient’s cell and a *Drosophila* model. Interactions between ALS-linked FUS and nucleoporins are associated with defects in the nucleocytoplasmic transport pathway in hiPSC-derived MNs harbouring FUS mutations. These species could be prevented from proper assembly into NPCs, leading to defects in the nucleocytoplasmic transport pathway. They also found that downregulation of Nup62 using RNA interference ameliorated the FUS–Nup62 interaction-induced toxic phenotype in *Drosophila* overexpressing the red fluorescent protein-tagged mutant FUS-P525L. This study strongly supports our findings and also suggests the need to investigate whether downregulation of Nup62 alters the phenotypes of the rodent *FUS*-ALS model in a future study.

Additionally, photoactivatable ribonucleoside-enhanced crosslinking and immunoprecipitation have revealed that FUS proteins target the mRNA of Lamin B1 and most nucleoporins, which might play important roles in mRNA maturation and gene regulation.^68^ These studies also revealed that *FUS* mutations showed a drastically altered binding pattern to these mRNAs, disrupting the RNA quality control system. Consistent with these results, we observed a decrease in the expression of the most nuclear lamina- and nucleoporin-linking genes in RNA sequencing of hiPSC-LMNs and the *FUS*^H517D^ mutation (Fig. 5I). These findings are supported by the aberrant staining of the G3BP1 stress granule, a key molecule of the RNA quality control system (Supplementary Fig. 4), and splicing regulator, TDP-43 (Supplementary Fig. 5). Therefore, we propose that the *FUS*^H517D^ mutation might directly dysregulate the expression of most nuclear lamina and nucleoporin RNAs, thereby disrupting the nuclear lamina and NPC.

Recent evidence reported that some nucleoporins are long-lived and do not turn over during the life of non-dividing cells, such as neurons.^69^ A subset of nucleoporins is oxidatively damaged in ageing cells, increasing nuclear permeability and leakiness. Therefore, the disruption of the nuclear lamina and nucleoporins is difficult or irreversible to correct, even in the early stages of ALS. If this hypothesis is correct, new therapeutic approaches should aim at overcoming the disruption of the nuclear lamina and nucleoporins at the earliest possible or presymptomatic stage, thereby preventing or delaying the neurodegeneration associated with *FUS*-ALS.

Furthermore, we detected DNA damage in MNs in the spinal cord of our mice and hiPSC-LMNs with *FUS*^H517D^ mutation (Supplementary Figs 7 and 8), supporting the findings of previous research in which DNA repair-related genes, including *TIMELESS*, were crucial for the molecular aetiology in iPSC-derived cell models with *FUS*^H517D^ mutation.^70,71^ Heterozygous *Fus*^WT/H509D^ mice show delayed but definite ALS-like motor phenotypes because *FUS*-H517D (basic amino acids, such as histidine, to acidic amino acids, such as aspartic acid) changes opposite charge, leading to reduced chaperoning by transportin-1 and aberrant liquid–liquid phase separation.^72,73^ Given that the nuclear lamina also plays an important role in DNA damage repair,^12,56^ nuclear lamina disruption might also cause DNA repair deficiency, in addition to the direct contribution of *FUS* mutation to DNA damage repair (Supplementary Figs 7 and 8). These findings are consistent with an autopsy study by Wang *et al*.^74^ showing increased DNA damage (γH2AX immunoreactivity) in the motor cortex of FALS patients harbouring R521C *FUS* mutation. They also reported that interaction of FUS and HDAC1 is crucial for DNA repair in both proliferating cells and primary neurons.

This study has some limitations and alternative interpretations that must be considered. First, we did not generate homozygous mice, which are expected to show enhanced phenotypes. *FUS*-ALS has an autosomal dominant genetic pattern in a pathological gain-of-function cascade, and the homozygous mutant FUS might lead to a partial loss of function owing to FUS mislocalization into the cytoplasm, making it difficult to interpret ALS phenotypes. Therefore, homozygous mice might be unsuitable for understanding the pathomechanism of practical ALS. Second, we did not assess the pathology of the forebrain and cognitive and behavioural impairments in *Fus*^WT/H509D^ mice. FUS is a key molecule of frontotemporal lobar degeneration (FTLD); however, FTLD prevalence in *FUS*-ALS is rare.^75-80^ Recent findings show FUS co-aggregation with the FET [FUS/Ewing sarcoma protein (EWS)/TATA-binding protein-associated factor 15 (TAF15)] protein and transportin-1 in *FUS*-FTLD but not in *FUS*-ALS linked with alterations in its arginine methylation, indicating distinct pathomechanisms of these diseases.^81^ However, the co-occurrence of ALS and FTLD in individuals with several FUS mutations has been reported.^59,75,82^ Shiihashi *et al*.^10,11^ reported that deleted NLS-FUS transgenic mice recapitulate ALS and FTD phenotypes. Future studies should assess FTD-like behavioural phenotypes and forebrain pathology and compare them with FUS arginine methylation in different regions.

## Conclusion

Conclusively, we established *Fus*^WT/H509D^ mice that exhibited a delayed but definite motor phenotype and nuclear lamina and NPC disruption. These findings were validated in patient-derived hiPSC-MNs with the same mutation and post-mortem spinal cord of patients with *FUS*-ALS. Combined with hiPSC-MNs and human samples, this mouse model might provide a more detailed understanding of ALS pathogenesis and development of treatments.

## Supplementary Material

awae224_Supplementary_Data

## Data Availability

The data sets generated and analysed in this study are available from the corresponding authors upon reasonable request. Untrimmed western blots are shown in Supplementary Fig. 9.
