# Supplementary material for: Multiple lines of evidence for disruption of nuclear lamina and nucleoporins in FUS amyotrophic lateral sclerosis
Source: Brain. 2024 Sep 23;147(11):3933–48. doi: 10.1093/brain/awae224 (PMC11684083; doi:10.1093/brain/awae224)
Supplement: awae224_Supplementary_Data [file awae224_supplementary_data.zip › brain-2023-01212-File009.pdf]

Supplementary Figure 1

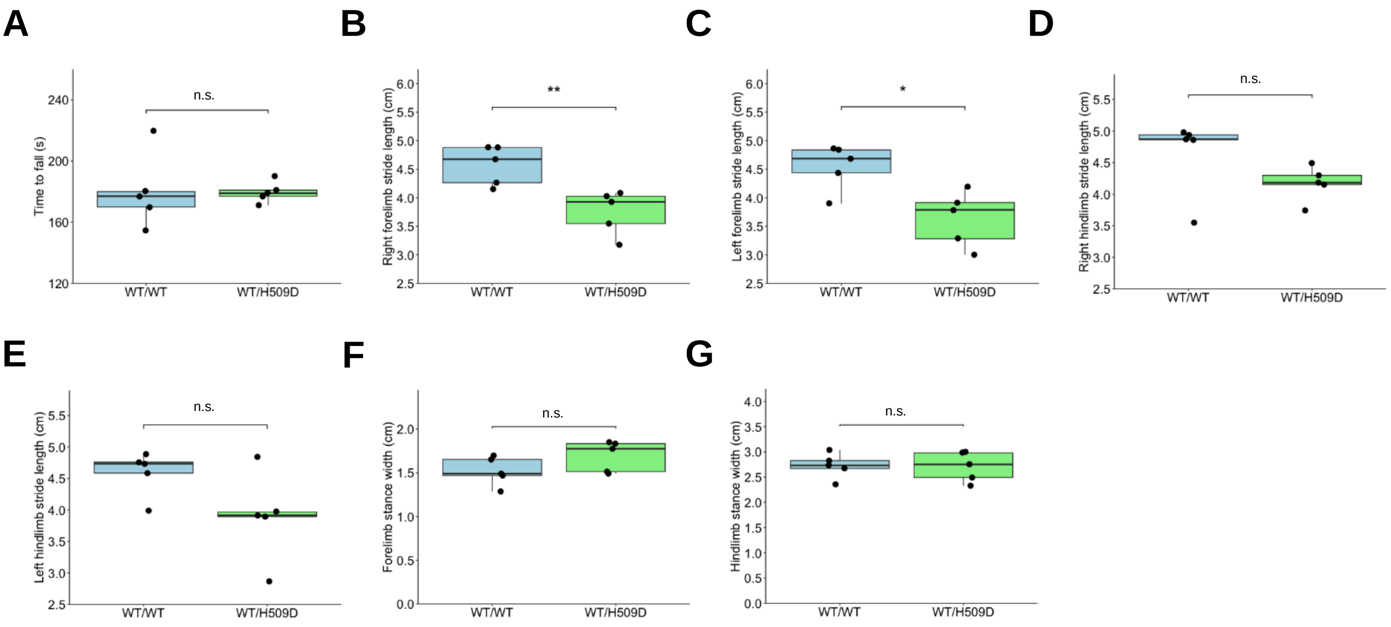

Supplementary Figure 1 Forelimb-predominant onset motor impairment in *Fus*$^{\mathrm{WT/H509D}}$ mice at 18 months

(**A**) Accelerating rotarod test results of wild-type and *Fus*$^{\mathrm{WT/H509D}}$ mice (18 months, $n = 5$ per genotype, male). Graphs represent quartile(boxes), 50th percentile(centerline) and range(whiskers). Student's *t*-test was used to calculate statistical significance. n.s. = no statistical significance. (**B**–**G**) DigiGait analysis of wild-type and *Fus*$^{\mathrm{WT/H509D}}$ mice (18 months, $n = 5$ per genotype, male). Graphs represent quartile(boxes), 50th percentile(centerline) and 1.5 times the interquartile range(whiskers). Student's *t*-test was used to calculate statistical significance. $*P < 0.05$, $**P < 0.01$. n.s. = no statistical significance.

Supplementary Figure 2

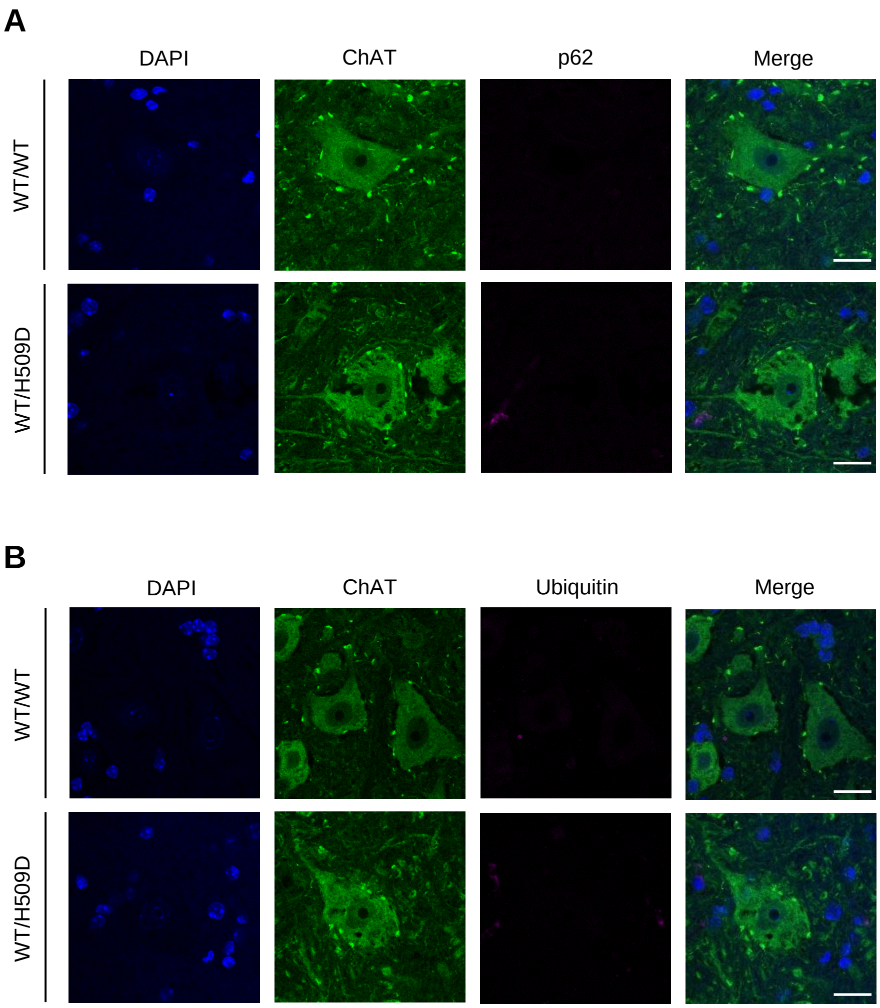

Supplementary Figure 2. No obvious p62 and ubiquitin-positive inclusion in the spinal cord of *Fus*$^{\mathbf{WT/H509D}}$ mice

(**A**) Immunofluorescence images of the spinal cord with ChAT (green) and p62 (magenta) staining in wild-type and *Fus*$^{WT/H509D}$ mice at 18 months. Images are representative of n = 3 experiments. Scale bar, 20 µm. (**B**) Immunofluorescence images of the spinal cord with ChAT (green) and ubiquitin (magenta) staining in wild-type and *Fus*$^{WT/H509D}$ mice at 18 months. Images are representative of n = 3 experiments. Scale bar, 20 µm.

Supplementary Figure 3.

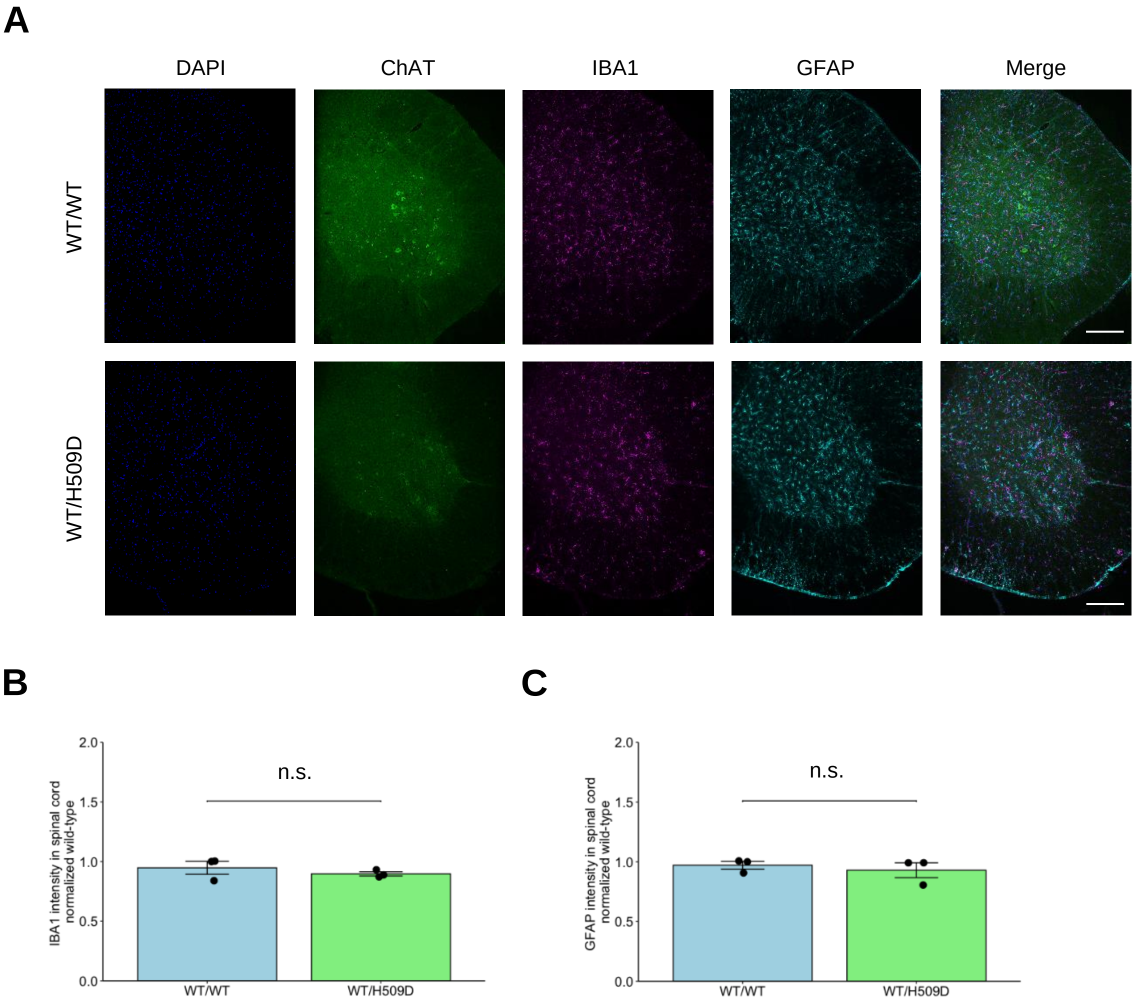

Supplementary Figure 3. No obvious increased intensity of IBA1 and GFAP in spinal cord of *Fus*$^{\mathbf{WT/H509D}}$ mice.

(A) Immunofluorescence images of spinal cord with ChAT (green) , IBA1 (magenta) and GFAP (cyan) staining in wild-type and *Fus*$^{WT/H509D}$ mice at 25 months of age. Images are representative of $n = 3$ experiments. Scale bar, 200 μm. (B, C) Quantification of IBA1 and GFAP intensity in the anterior horn of spinal cord. Student's t test was used to calculate statistical significance. n.s. = no statistical significance.

Supplementary Figure 4.

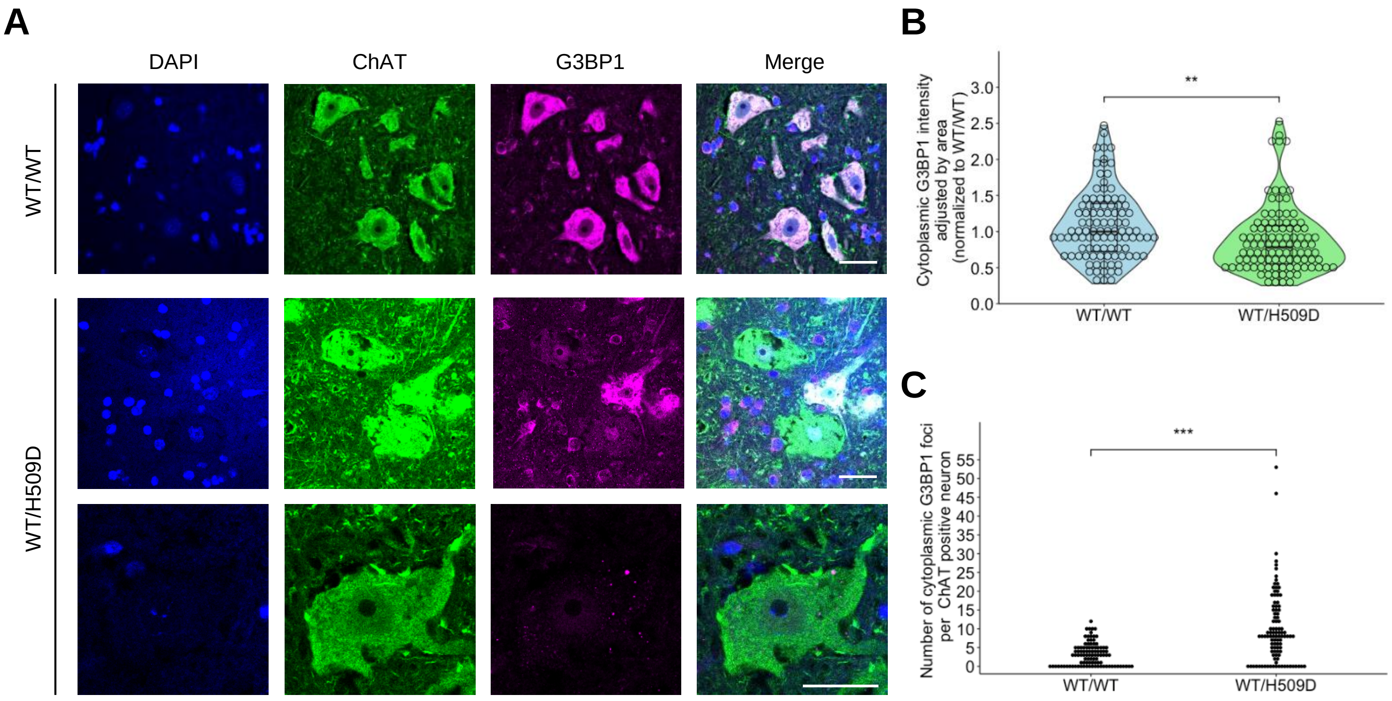

Supplementary Figure 4. Decreased expression of G3BP1 and disturbance of stress granules in the spinal cord of *Fus*$^{\mathrm{WT/H509D}}$ mice

(**A**) Immunofluorescence images of the spinal cord with ChAT (green) and G3BP1 (magenta) staining in wild-type and *Fus*$^{\mathrm{WT/H509D}}$ mice at 18 months. Images are representative of $n = 3$ experiments. Scale bar, 20 µm. (**B**) Quantification of the intensity of cytoplasmic G3BP1 in ChAT-positive cells in the spinal cord ($n = 3$ per genotype, 100 cells wild-type; 102 cells *Fus*$^{\mathrm{WT/H509D}}$, male). Graphs represent quartile(boxes), 50th percentile(centerline) and 1.5 times the interquartile range(whiskers). Student's t-test was used to calculate statistical significance. $^{**}P < 0.01$. (**C**) Quantification of the number of cytoplasmic G3BP1 foci per ChAT-positive cells in the spinal cord ($n = 3$ per genotype, 100 cells wild-type; 102 cells *Fus*$^{\mathrm{WT/H509D}}$, male). Student's t-test was used to calculate statistical significance. $^{***}P < 0.001$. ChAT = choline acetyltransferase.

Supplementary Figure 5.

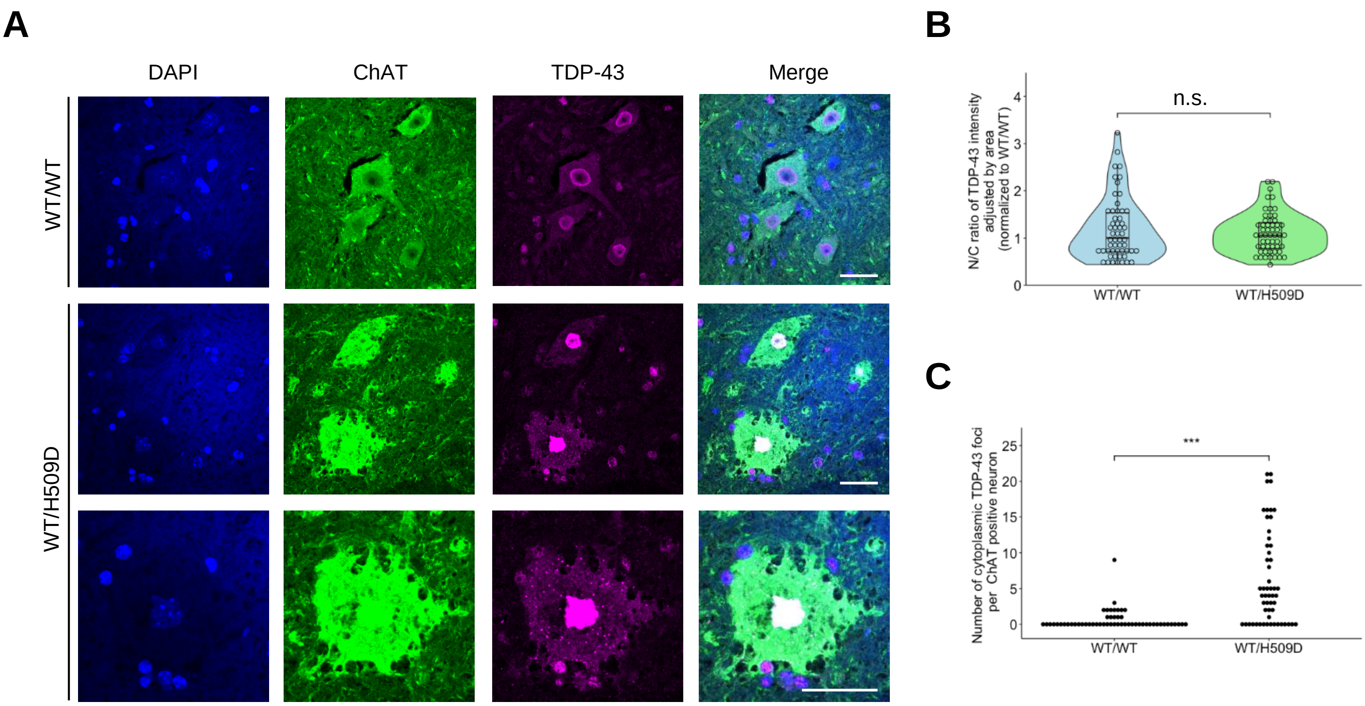

Supplementary Figure 5. Disturbance of cytosolic TDP-43 in spinal cord of *Fus*$^{WT/H509D}$ mice.

(A) Immunofluorescence images of spinal cord with ChAT (green) , TDP-43 (magenta) and staining in wild-type and *Fus*$^{WT/H509D}$ mice at 18 months of age. Images are representative of n = 3 experiments. Scale bar, 20 µm. (B) Quantification of N/C ratio of TDP-43 intensity in ChAT-positive neurons in the spinal cord (n = 3 per genotype, 56 cells wild-type; 54 cells *Fus*$^{WT/H509D}$, male). Graphs represent quartile(boxes), 50th percentile(centerline) and 1.5 times the interquartile range(whiskers). Student's t-test was used to calculate statistical significance. n.s. = no statistical significance. (C) Quantification of the number of cytoplasmic TDP-43 foci per ChAT-positive neuron in the spinal cord (n = 3 per genotype, 56 cells wild-type; 54 cells *Fus*$^{WT/H509D}$, male). Student's t-test was used to calculate statistical significance. ***$P < 0.001$. ChAT = choline acetyltransferase.

Supplementary Figure 6.

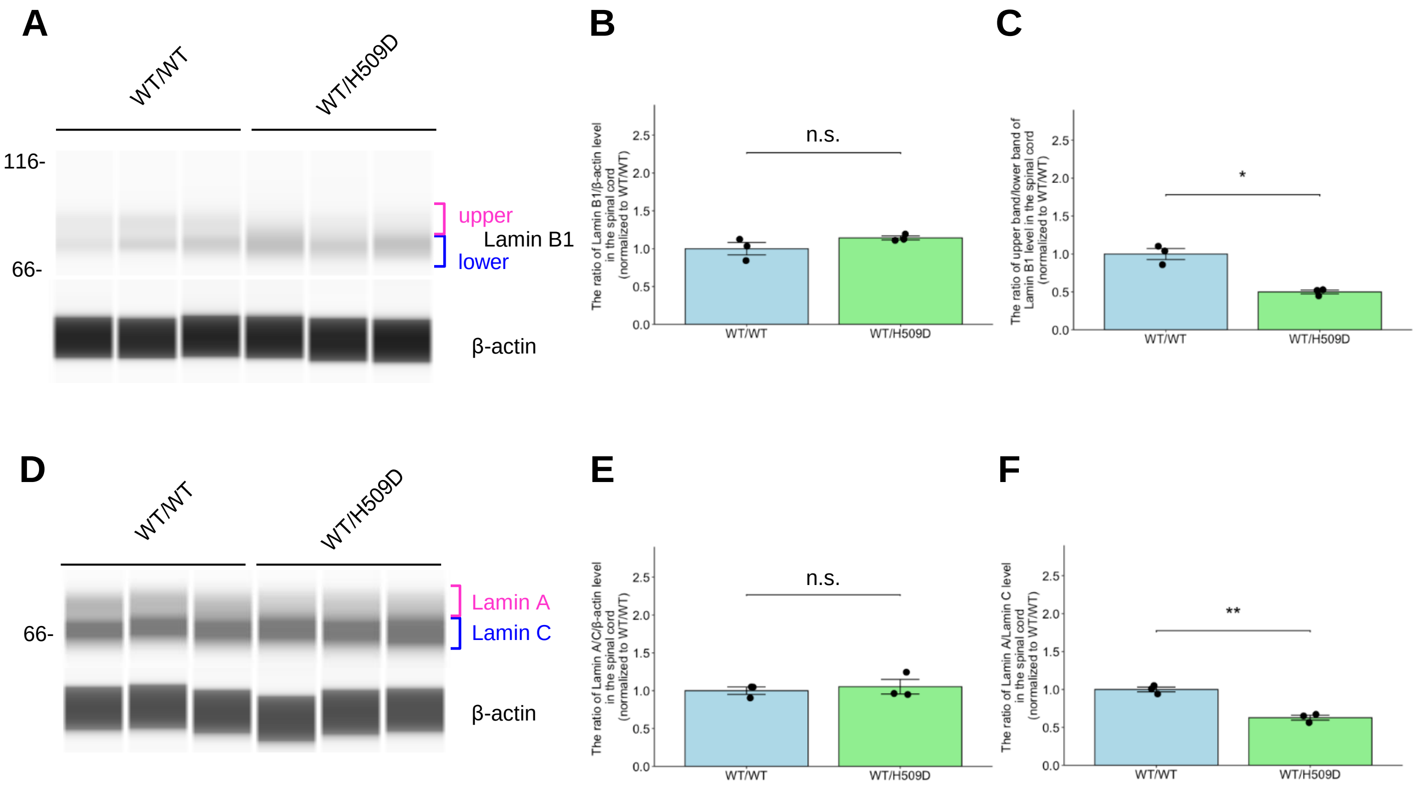

Supplementary Figure 6. Imbalanced Lamin B1 and Lamin A/C in the spinal cord of *Fus*$^{WT/H509D}$ mice.

(A) Western blot analysis of spinal cord with Lamin B1 in wild-type and *Fus*$^{WT/H509D}$ mice at 25 months of age. (B) Quantification of total Lamin B1 level (magenta and blue) in the spinal cord. Student's t test was used to calculate statistical significance. n.s. = no statistical significance. (C) Quantification of the ratio of upper band (magenta) to lower band (blue) of Lamin B1 level in the spinal cord. Student's t test was used to calculate statistical significance. * $P < 0.05$. (D) Western blot analysis of spinal cord with Lamin A/C in wild-type and *Fus*$^{WT/H509D}$ mice at 25 months of age. (E) Quantification of total Lamin A/C level in the spinal cord. Student's t test was used to calculate statistical significance. n.s. = no statistical significance. (F) Quantification of the ratio of Lamin A (magenta) to Lamin C (blue) level in the spinal cord. Student's t test was used to calculate statistical significance. * * $P < 0.01$.

Supplementary Figure 7.

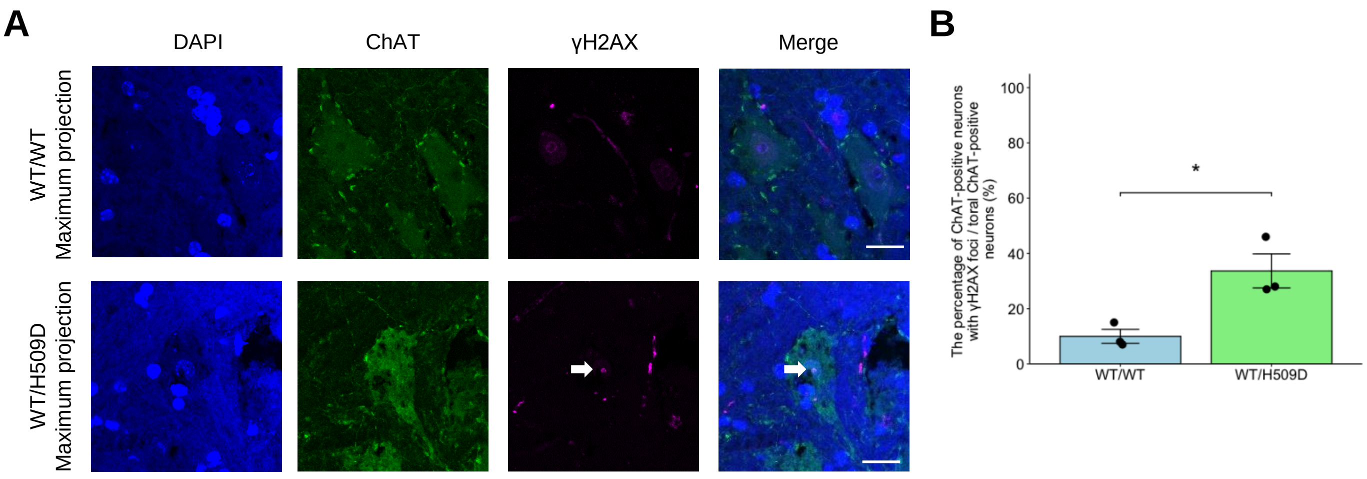

Supplementary Figure 7. Increased DNA damage in *Fus*$^{WT/H509D}$ mice

(**A**) Immunofluorescence images of the spinal cord with ChAT (green) and γH2AX (magenta) staining in wild-type and *Fus*$^{WT/H509D}$ mice at 18 months. Images are representative of $n = 3$ experiments. White arrows show γH2AX foci. Scale bar, 20 μm. (**B**) Quantification of the percentage of ChAT-positive neurons with γH2AX foci/total number of ChAT-positive neurons in the spinal cord (Overlaid bar graph and dot plot). Data are presented as mean ± SEM. Student's t test was used to calculate statistical significance. *$P < 0.05$. ChAT = choline acetyltransferase, SEM = standard error of the mean.

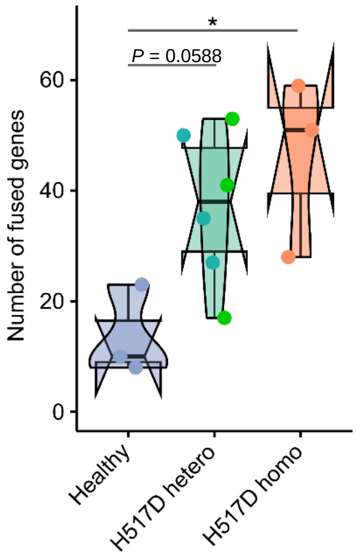

Supplementary Figure 8. Increased the number of fusion gene for hiPSC-LMNs in FUS-H517D mutation.

Results of the number of fusion gene in Healthy-, H517D hetero-1 and 2-, and H517D homo-LMNs. Graphs represent quartile(boxes), 50th percentile(centerline) and 1.5 times the interquartile range(whiskers). Dunnett's test was used to calculate statistical significance. * $P < 0.05$.

Supplementary Figure 9.

Fig. 1D

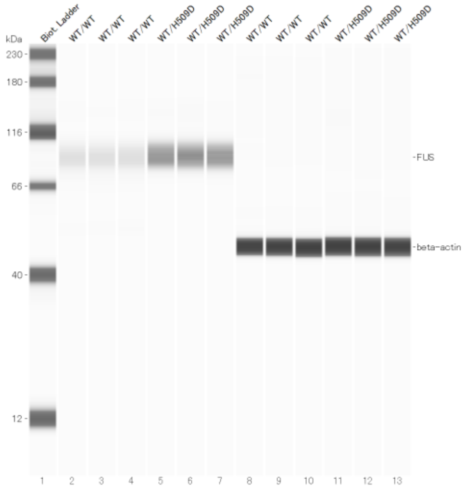

Supplementary Figure 6A

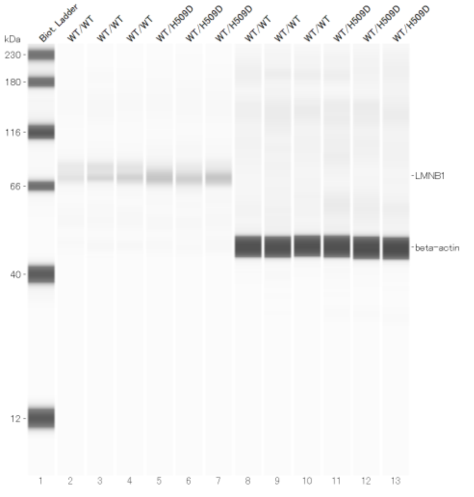

Supplementary Figure 6D

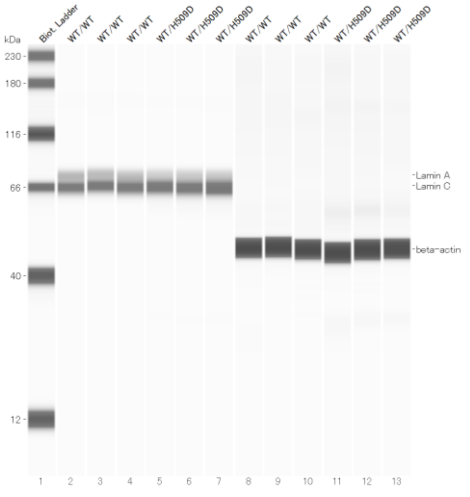

Supplementary Figure 9. Uncropped western blot.
