# Supplementary material for: Multiple lines of evidence for disruption of nuclear lamina and nucleoporins in FUS amyotrophic lateral sclerosis
Source: Brain. 2024 Sep 23;147(11):3933–48. doi: 10.1093/brain/awae224 (PMC11684083; doi:10.1093/brain/awae224)
Supplement: awae224_Supplementary_Data [file awae224_supplementary_data.zip › brain-2023-01212-File010.pdf]

Supplementary Table 1

Clinical Data					
Case	Age, Years	Sex	Post mortem time (h)	Diagnosis	Duration of ALS, Months
ALS					
1	65	F	2	SALS	41
2	75	M	8	SALS	NA
3	76	F	8	SALS	NA
4	64	M	4	SALS	94
5	61	M	3	SALS	18
6	70	F	5	SALS	40
7	49	F	NA	FALS(FUS-R521L)	12
8	40	M	NA	FALS(FUS-R521L)	108
Control					
1	38	M	2.5	Lung cancer	
2	66	M	8	Malignant melanoma	
3	74	M	3	Acute myocardial infarction	
4	48	F	15.5	Cardiac angiosarcoma	
5	82	M	17	Acute myocardial infarction	
6	76	F	8.5	Multiple cerebral infarction	

ALS, amyotrophic lateral sclerosis; F, female; FALS, familial ALS; M, male; NA, not applicable; SALS, sporadic ALS.

Supplementary Table 2

	FUS$^{WT/WT}$	*FUS*$^{WT/H517D}$	*FUS*$^{H517D/H517D}$	Freq.
Pattern 1	A	A	A	33193
Pattern 2	B	A	A	3446
Pattern 3	A	A	B	463
Pattern 4	A	B	A	145
Pattern 5	A	B	C	160
